# Alcohol Consumption and DNA Methylation in a Mediterranean Cohort: A Focus on Oxidative Stress and Aging Biomarkers

**DOI:** 10.3390/antiox15020197

**Published:** 2026-02-02

**Authors:** Oscar Coltell, Eva M. Asensio, José V. Sorlí, Rebeca Fernández-Carrión, Carolina Ortega-Azorín, Rocío Barragán, Alejandro Perez-Fidalgo, Olga Portolés, Jose M. Ordovas, Dolores Corella

**Affiliations:** 1 Department of Computer Languages and Systems, Universitat Jaume I, 12071 Castellón, Spain; oscar.coltell@uji.es; 2 Centro de Investigación Biomédica en Red (CIBER) “Fisiopatología de la Obesidad y Nutrición”, Instituto de Salud Carlos III, 28029 Madrid, Spain; eva.m.asensio@uv.es (E.M.A.); rebeca.fernandez@uv.es (R.F.-C.); carolina.ortega@uv.es (C.O.-A.); rocio.barragan@uv.es (R.B.); olga.portoles@uv.es (O.P.); jose.ordovas@tufts.edu (J.M.O.); 3 Department of Preventive Medicine and Public Health, School of Medicine, University of Valencia, 46010 Valencia, Spain; 4 Division of General Medicine, Department of Medicine, Columbia University Irving Medical Center, New York, NY 10032, USA; 5 University Clinic Hospital of Valencia, INCLIVA Biomedical Research Institute, 46010 Valencia, Spain; japfidalgo@msn.com; 6 Centro de Investigación Biomédica en Red (CIBER) “Cáncer”, Instituto de Salud Carlos III, 28029 Madrid, Spain; 7 Nutrition and Genomics Laboratory, Jean Mayer—US Department of Agriculture Human Nutrition Research Center on Aging, Tufts University, Boston, MA 02111, USA; 8 Instituto Madrileño de Estudios Avanzados en Alimentación (IMDEA) Nutrition Institute, Campus de Excelencia Internacional, Universidad Autónoma de Madrid (UAM) + Consejo Superior de Investigaciones Científicas (CSIC), 28049 Madrid, Spain

**Keywords:** alcohol, methylation, epigenomics, reactive oxygen species, biomarkers, telomere length, aging, antioxidant defense, Mediterranean diet, oxidative stress

## Abstract

There is considerable interest in the connection between alcohol-induced oxidative stress, DNA methylation, antioxidants, and accelerated aging across diverse populations. Nevertheless, self-reported alcohol consumption is prone to bias, and objective biomarkers of alcohol intake are needed. Our aims were to investigate the performance of an epigenomic biomarker of alcohol consumption in a Mediterranean population using self-reported data and the biomarker gamma-glutamyl transferase (GGT); to examine the effects of alcohol (self-reported and biomarker-assessed) on epigenome-wide methylation; to analyze the association between alcohol (self-reported and biomarker-assessed) and telomere length and other aging biomarkers; and to explore the modulating effect of the Mediterranean diet (MedDiet). We performed blood epigenome-wide methylation studies (EWAS) in a Mediterranean cohort (aged 55–75 years). Self-reported alcohol consumption and MedDiet were assessed by questionnaires. A replication cohort (cohort 2) from the same area was also analyzed. For both cohorts, the DNA methylation-based biomarker (450-CpGs) was computed alongside epigenetic clocks for the following biological age acceleration metrics: DNAm telomere length, GrimAgeAcceleration, PhenoAgeAcceleration, and CausalityAgeYing (cohort 1). The association between the epigenomic biomarker and self-reported alcohol consumption was significant (*p* < 0.001) in both cohorts, but modest. However, the association was stronger when predicting high alcohol intake (AUC: 0.76; 95%CI: 0.65–0.86; *p* < 0.0001). In the EWAS, the hit (cg06690548-*SLC7A11*, in a cystine transporter that enhances glutathione production for antioxidant defense) was shared among the self-reported alcohol consumption, GGT, and the epigenomic biomarker, with alcohol linked to hypomethylation. We detected differential methylation in pre-selected oxidative stress-related genes. Enrichment analysis revealed “Rap1 signaling pathway” as the hit (*p* < 0.00001). High self-reported alcohol consumption and the epigenomic biomarker were associated with shorter telomere length (*p* < 0.05) in cohort 1. Additionally, a modulation by Mediterranean diet adherence was hypothesized. No significant associations were found between self-reported alcohol intake and the other aging biomarkers; however, the epigenomic score was directly associated with GrimAge, PhenoAge and CausAgeYing biomarkers in cohort 1 (*p* < 0.001), and two were replicated in cohort 2. In conclusion, alcohol intake has an impact on DNA methylation at the epigenome-wide level in this Mediterranean population, replicating the main hits from other populations and validating the epigenomic biomarker for intake, although improvement is needed. Moreover, several associations with aging biomarkers were observed.

## 1. Introduction

Alcohol consumption constitutes an important global health concern [1]. Numerous diseases have been associated with alcohol intake, especially at higher levels of consumption [2,3,4,5,6,7]. However, the type of alcoholic beverage and the potential protective effects of light consumption compared to abstinence continue to be topics of active discussion, particularly in regard to their J- or U-shaped association with various cardiovascular diseases and related factors [8,9,10,11,12].

Currently, there is a growing interest in the relationship between alcohol consumption and aging [13,14,15]. Aging has been defined as the time-related deterioration that occurs in an organism at all levels, ranging from the molecular and cellular to the physiological and functional [16]. The process of aging is propelled by key features, so-called “hallmarks of aging.” Twelve hallmarks of aging have been proposed [17], including: telomere attrition, epigenetic alterations, deregulated nutrient-sensing, mitochondrial dysfunction, genomic instability, altered intercellular communication, chronic inflammation, and cellular senescence. Alcohol consumption, through increased oxidative stress, reactive oxygen species, and other mechanisms, has been implicated in all of these hallmarks, although its relevance varies among them [18,19,20,21,22,23,24,25,26,27,28,29,30]. These pleiotropic effects of alcohol depend on the amount and duration of consumption but generally begin with the generation of highly reactive molecules, such as reactive oxygen species (ROS) and reactive nitrogen species (RNS) in hepatocytes [31]. These ROS and RNS can disrupt antioxidant defense systems, resulting in increased lipid peroxidation, DNA damage, and impaired function of mitochondria and other organelles and processes. Furthermore, oxidative stress contributes to the activation of inflammatory pathways, thereby exacerbating the harmful effects [32] related to specific diseases [33]. One of the hallmarks of aging extensively investigated is the effect of alcohol on telomere length. Telomeres are the protective ends of linear chromosomes that decrease in length over the duration of an individual’s life [34]. They consist of tandem repeats of the TTAGGG DNA sequence bound by a six-protein complex known as shelterin [34,35]. Telomeres preserve genome integrity and enable cell proliferation [36]. Shorter telomere length has been associated with increased risk of several diseases and mortality [37,38,39,40]. Generally, research involving individuals with alcohol use disorder has identified inverse associations between alcohol consumption and telomere length [41,42]. Nevertheless, the findings regarding alcohol’s influence on telomere length among individuals from the other groups have been less consistent [27,43,44,45,46]. This has been emphasized by methodological difficulties in quantifying telomere length in humans, due to the diverse approaches available [47], as well as the issues in accurately assessing alcohol consumption in epidemiological studies [11]. Some Mendelian randomization studies, which utilize genetic variables as proxies for alcohol consumption, have identified significant associations with shorter telomere length [48,49], but these studies have also been subject to criticism, requiring further research.

Among the other hallmarks of aging, epigenetic alterations are highly relevant. There are several types, although the best-known is DNA methylation [50,51]. It consists of the transfer of a methyl group from S-adenosyl methionine to cytosine residues at the carbon 5 position (5-methylcytosine [5-mC]), primarily taking place within the context of cytosine-phosphate-guanine (CpG) dinucleotides [51]. Interestingly, DNA methylation represents a dynamic epigenetic modification and can be modified by specific interventions [52]. These DNA methylation markers serve as key regulators of essential functions and are associated with the risk of several diseases [50,51,52,53]. The effect of alcohol consumption on DNA methylation is still not well understood. Although several epigenome-wide association studies (EWAS) have been conducted to identify the CpG loci most strongly associated with alcohol consumption [54,55,56,57,58,59,60,61,62,63,64,65,66], the results have generally been heterogeneous, and further research is still needed. Despite the early stage of the EWAS results, it is understood that alcohol influences DNA methylation via several pathways [67,68,69,70]. These mechanisms include (a) induction of oxidative stress, whereby ROS and RNS interfere with methylation status at CpG sites; (b) inhibition of folate metabolism, which diminishes the production of the methyl donor S-adenosylmethionine (SAM), consequently reducing the activity of DNA methyltransferases (DNMTs); and (c) direct modulation of DNMTs and ten-eleven translocation (TET) enzymes [68]. Although several methylation loci have been replicated across multiple studies, the consistency of alcohol’s effects on specific CpGs is not as high as the high consistency reported for tobacco’s effects [71,72]. Furthermore, several EWAS did not assess alcohol consumption in grams per day (g/d) but instead classified individuals based on whether or not they had alcohol use disorders. In general, in the EWAS for alcohol, the most frequently repeated association was hypomethylation of the cg06690548 in the *SLC7A11* (Solute Carrier Family 7 Member 11) gene, which provides a crucial defense against oxidative stress by importing cystine for glutathione (GSH) production. However, it has not always been the most significant. Since self-reported alcohol consumption is known to be subject to recall bias or social desirability bias [73], it is necessary to have more objective biomarkers of alcohol consumption. Among them, traditional biomarkers such as gamma-glutamyl transferase (GGT) are commonly used as indirect indicators; however, they exhibit limited specificity [74]. Other emerging biomarkers, such as ethyl glucuronide and phosphatidylethanol, exhibit greater specificity but vary in their detection windows [75]. Moreover, methylation-based biomarkers have been proposed to improve the limitations [76], but this is still an emerging area of active research. In terms of methylation-based biomarkers, the epigenomic risk score based on the methylation of 450 CpG sites is particularly noteworthy [77]. This biomarker was proposed by McCartney et al. [77] for habitual alcohol consumption. Although this methylation-based score has been previously used in other populations [78,79], its validity in the Spanish Mediterranean population is unknown.

Similarly, DNA methylation has represented a significant advancement in measuring various aging biomarkers [80,81]. Although other genomic, metabolomic, transcriptomic, and proteomic markers exist for measuring aging [82], epigenomic biomarkers based on DNA methylation have shown greater validity and reliability than others [83,84]. Several types of aging biomarkers based on the methylome, also so-called “epigenetic clocks,” have been proposed [85,86]. The first generation of clocks was trained to measure chronological age [87]. The second-generation clocks were trained to measure morbility and mortality phenotypes; two of the most widely used have been GrimAge and PhenoAge [88,89]. Recently, a clock based on Mendelian randomization, called CausAgeYing [90], has been proposed. Likewise, a new methylation-based biomarker of telomere length was developed [91], which we have previously validated in this Mediterranean population [92], and which recent studies indicate has better predictive value than classical measures [93].

While limited research has investigated the connection between alcohol use and aging biomarkers, current findings suggest a link between higher alcohol consumption and accelerated biological aging. However, the results are inconsistent, and further research is necessary [56,58,88,89,94,95,96,97,98,99]. Furthermore, no prior study has been undertaken in a Spanish Mediterranean population. Moreover, oxidative stress can be mitigated by antioxidants. The Mediterranean diet is recognized for its rich antioxidant content [100,101], which may modulate the physiological impact of alcohol consumption. Consequently, the modulating influence of varying degrees of adherence to the Mediterranean diet warrants investigation. Therefore, our objectives were (1) to investigate the performance of an epigenomic biomarker for alcohol intake in this Mediterranean population, also using the conventional GGT; (2) to examine the effects of alcohol consumption (self-reported and biomarkers) on the epigenome-wide methylation in this population also investigating methylation in pre-selected oxidative stress-related genes and conducting pathway analysis, as well as exploring some modulating effects of the Mediterranean diet adherence; and (3) to analyze the association between alcohol consumption (assessed through self-reporting and biomarkers) with telomere length, as well as with other biomarkers of aging (GrimAge, PhenoAge and CausAgeYing) and to explore the potential modulation by the Mediterranean diet.

## 2. Materials and Methods

### 2.1. Study Population and Replication Sample

We carried out a cross-sectional study in a Mediterranean population consisting of 414 participants (white Southern European individuals) aged 55 to 75 years (cohort 1), recruited from the Valencia region (Spain) as part of the PREDIMED-Plus-Valencia study [102]. This site was among the field sites for the multicenter PREDIMED-Plus trial [103] and is the sole location with epigenome-wide methylation data available. Participants were community-dwelling men and women with metabolic syndrome and free of cardiovascular disease at baseline, recruited from several primary health care centers in Valencia. Detailed characteristics of the study design and inclusion/exclusion criteria have been published [104]. This was considered the main cohort. In addition, a replication cohort (cohort 2) was analyzed for some associations.

Participants in the replication cohort were white Southern European men and women recruited from the same region and aged 55–80 years. These subjects were participants of the multicenter PREDIMED-Valencia study [105], one of the sites of the multicenter PREDIMED study [106]. In this cohort, individuals were at high cardiovascular risk but free of cardiovascular disease. Detailed characteristics of the study design and inclusion/exclusion criteria were published in [106]. We analyzed n = 150 participants with epigenome-wide methylation data available at baseline in the Valencia site. In both cohorts, participants provided written informed consent, and study protocols and procedures were approved in compliance with the Helsinki Declaration by Valencia University’s Human Research Ethics Committee (ethical approval codes H1373255532771, 15 July 2013; H1509263926814, 6 November 2017; and 1575541280191, 5 December 2019).

### 2.2. Anthropometric, Clinical, and Biochemical Variables at Baseline

In cohort 1, anthropometric data and blood pressure were assessed by qualified personnel according to the PREDIMED-Plus operating protocol, and body mass index (BMI) was computed [104]. Blood pressure was measured using a validated semiautomatic oscillometer (Omron HEM-705CP, OMRON Healthcare Europe B.V., Hoofddorp, The Netherlands). Fasting blood samples were collected, and plasma glucose, total cholesterol, HDL-C, LDL-C, triglycerides, GGT, alanine aminotransferase (ALT), aspartate aminotransferase (AST), and a complete blood count—including total leukocyte counting as well as the types of white blood cells (neutrophils, eosinophils, basophils, monocytes, and lymphocytes)—was determined as previously reported using automated hematology analyzers [72,107]. Medication use was assessed at baseline by a validated questionnaire, and type 2 diabetes (hereinafter referred to as diabetes) was defined as a previous clinical diagnosis of diabetes, HbA1c levels ≥ 6.5%, or use of anti-diabetic medication [103]. For the replication cohort (cohort 2), the anthropometric data, blood pressure, fasting blood samples, biochemical determinations, and leukocyte counts were obtained by a similar procedure at the same site [105,108]. Likewise, medications and type 2 diabetes were assessed using the same methodology [105].

### 2.3. Self-Reported Alcohol Intake, Adherence to the Mediterranean Diet, and Other Lifestyle Factors

Self-reported alcohol intake in the PREDIMED-Plus-Valencia study was assessed at baseline with a validated 143-item food-frequency questionnaire (FFQ), which included nine questions on consumption of different alcoholic beverages (wine, beer, and spirits) daily, per week, or per month over the previous year [104,109]. The specific items for self-reported alcohol consumption included wine (red wine [aged and young], other wine [white, rosé, muscatel, and cava]), beer, and spirits (liquor and whiskey). For the calculation of pure alcohol content in g/d, standard sizes of each type of beverage in the FFQ were computed as previously reported [109]. In the replication cohort (PREDIMED-Valencia), self-reported alcohol intake was assessed with a similar FFQ (only with 137 items, but with no difference in the items for alcoholic beverages) [109,110]. Further, alcohol consumption was categorized into four drinking categories according to different cut-off points, taking into account sex differences. First, for international comparability with previous works, we used the cut-off points published by Lui et al. [63]. The continuous variable was first categorized into four drinking categories: ‘non-drinkers’ (0 g/d); light drinkers (0 < g/d ≤ 28 in men and 0 < g/d ≤ 14 in women); at risk-drinkers (28 < g/d < 42 in men and 14 < g/day < 28 in women); heavy and drinkers (≥42 g/d in men and ≥28 g/d in women). Second, taking into account that this was a population of mostly low-to-moderate alcohol users, the number of subjects in the highest international categories was very small, and we also categorized alcohol in another variable with small cut-off points using the alcohol content (10 g of pure alcohol) of the standard drinks in this country [111,112]: non-drinkers (alcohol = 0); light drinkers (<20 g/d in men and <10 g/d in women); moderate drinkers (20–40 g/d in men and 10–20 in women); and heavy drinkers (≥40 g/d in men and ≥20 g/d in women). For statistical analysis, alcohol consumption as a continuous variable was square-root transformed.

In cohort 1, Mediterranean diet adherence was assessed by a validated Mediterranean diet adherence score (MEDAS), which includes 17 items [113] and is an updated version of the previously validated MEDAS-14 score [114]. The scale was scored with 1 point for each item adhering to the Mediterranean diet and 0 points for each item that did not. A higher score (from 0 to 17) indicated higher Mediterranean diet adherence. Next, the score was categorized into two groups based on the mean: low (from 0 to 8 points) or high (from 9 to 17 points) adherence. Additionally, it is important to note that the MEDAS-17 score also included an item regarding moderate wine consumption; thus, for further analysis, we removed this item and calculated a new score reduced to 16 points. This score was categorized as low adherence (0–8) and high adherence (9–16) for some analyses, as indicated. In cohort 2, Mediterranean diet adherence was assessed using the MEDAS-14 score only [114]. Tobacco smoking and education were assessed by the same general questionnaire in both cohorts [102,105]. Physical activity in both cohorts was assessed by validated questionnaires (analyzing total leisure-time physical activity-related energy expenditure) [115,116].

### 2.4. DNA Isolation and DNA Methylation Analysis

DNA isolation and methylation analyses in the main and replication cohorts were carried out using the same protocol. Genomic DNA was isolated from blood as reported in [102,105]. DNA was quantified utilizing PicoGreen (Invitrogen Corporation, Carlsbad, CA, USA). Only samples with high-quality DNA were included for epigenome-wide methylation profiling. We conducted methylation analysis on all participants in the main cohort with available DNA. In the replication cohort, we analyzed only a subset due to cost constraints. For both cohorts, we used the Infinium HumanMethylationEPIC BeadChip (850 K) array (Illumina, San Diego, CA, USA) for methylation profiling, which interrogates over 850,000 CpG sites. The positions of the samples on the microchips were randomized to minimize batch effects [72]. Further processing of the arrays was carried out at the Human Genomics Facility, Erasmus MC, Rotterdam. Bisulfite conversion and hybridation were performed according to the manufacturer’s protocol [72]. Microarrays were scanned with an Illumina HiScan system. Quality control procedures were implemented to assess the quality and reliability of the methylation data using the Minfi (Bioconductor; 1.52.0), Meffil (GitHub; 1.3.1), and ewastools (CRAN; 1.7.2) R packages (version 4.4 or higher) [117,118]. We identified samples that failed or exhibited suboptimal control metrics, including inadequate bisulfite conversion, poor hybridization, and low call rates. A total of 414 samples successfully underwent quality control and were used in further analysis for the EWAS in cohort 1. In cohort 2, a total of 150 samples achieved satisfactory quality control. Additional DNA assessments, data normalization, and filtering procedures were conducted utilizing the Partek^®^ Genomics Suite^®^ (version 7.20.0831; St. Louis, MO, USA) [119]. Probes originating from the X and Y chromosomes were filtered and subsequently excluded. Functional normalization [120] and normal-exponential out-of-band (NOOB) background correction were performed [121]. Beta-values (ranging from 0 to 1) were computed for the CpG sites. Next, beta-values were converted to M-values for statistical testing as follows: M-value = log_2_ (beta/(1 − beta)), taking into account that M-values have higher homoscedasticity compared with beta-values [122]. Further, we used beta-values for direct biological interpretation (corresponding to the percentage of a CpG site that is methylated).

### 2.5. Epigenomic Score for Alcohol Consumption

The DNA methylation-derived alcohol score was computed using the 450-CpG sites and coefficients published by McCartney et al. [77]. Table S1 presents 30 CpGs included in this score (comprising the top 15 CpGs with positive weights and the 15 CpGs with the most important negative weight, listed according to beta values in the score). It also indicates the annotated gene. The complete list, of the epigenomic biomarker of alcohol consumption, can be found in the published paper [77]. The 450-CpG score was calculated both for cohort 1 and cohort 2 by multiplying each set of beta coefficients against the DNA methylation levels obtained in the normalized epigenome-wide methylation arrays for each cohort. This analysis was performed using the online Horvath epigenetic age calculator [123] for cohorts 1 and 2, which also included the estimation of this epigenomic score for alcohol intake. Furthermore, for quality control, in cohort 1, we independently calculated the 450-CpG methylation-derived alcohol score using the Biolearn platform (0.8.0, 2025) [124]. The correlation between the two platforms was perfect (r^2^ = 1), indicating excellent quality control in the computation. We used the epigenomic score derived from the online Horvath epigenetic calculator for subsequent statistical analysis, noting that it was calculated in both cohort 1 and in cohort 2. Furthermore, we assessed the quality of the methylation computations using the gold standard, and all the samples passed the criterion corSampleVSgoldstandard at >0.90, as previously detailed [72].

### 2.6. Statistical Analysis, EWAS, and Calculation of Aging Biomarkers

Descriptive statistics were performed. Chi-square tests were used to compare proportions, and Student *t*-tests and ANOVA tests were applied to compare crude means of continuous variables. Square-root transformation of self-reported alcohol consumption as a continuous variable was carried out. GGT and triglyceride concentrations were transformed using the natural logarithm for statistical testing. We analyzed the association between the biomarkers of alcohol consumption and self-reported alcohol consumption using general linear models adjusted for covariates in both cohort 1 and in the replication cohort. Pearson correlation, heat maps, and scatter plots were displayed.

Also, we tested in this population (cohort 1 and cohort 2) the predictive value of the 450-CpG methylation-based score for detecting high alcohol consumption using receiver operative curves (ROCs). The area under the curve (AUC), its 95% confidence interval (CI), and *p*-values were computed for the epigenomic biomarker in each cohort. We checked the performance of these classification models using SPSS Statistics for Windows Ver. 26 (IBM Corp., Armonk, NY, USA). A *p*-value < 0.05 (two-sided) was considered statistically significant.

To estimate the association between self-reported alcohol consumption and epigenome-wide DNA methylation in cohort 1 (the sample size in cohort 2 was underpowered for EWAS), we performed several analyses, including additional adjustments for covariates. In addition to the raw model, other multivariable models were sequentially adjusted for sex, age, BMI, diabetes, smoking batch effect, and leukocyte cell counts. Instead of using estimated leukocyte cell counts [125], we employed directly measured cell counts (neutrophils, eosinophils, basophils, monocytes, and lymphocytes) after checking the potential multicollinearity [72,126]. After verifying the similar estimates obtained in each step, we only presented the results corresponding to the multivariable regression model adjusted for all covariates. Additional adjustments of the multivariable models for the EWAS were fitted when indicated. CpGs were annotated to the corresponding genes, and *p*-values and the partial regression coefficients for each CpG site were estimated. To account for the correction for multiple comparisons, we set the *p*-value cut-off for EWAS statistical significance at *p* < 9 × 10^−8^, according to Mansell et al. [127], when using the EPICv1.0 methylation array. Also, we used the standard level of suggestive statistical significance in the EWAS (*p* < 1 × 10^−5^). Manhattan plots of the adjusted EWAS model were computed in R and depicted. For quality control, quantile–quantile (Q-Q) plots comparing the expected and observed *p*-values were performed in the R-statistical environment. For self-reported alcohol consumption, we also analyzed the differential methylation of pre-selected oxidative stress-related genes related to alcohol use [21,31]. We focused on 37 genes from the mentioned references [21,31]: *ACSS2*, *ADH1A*, *ADH1B*, *ADH1C*, *ADH4*, *ADH5*, *ADH6*, *ADH7*, *ALDH1A1*, *ALDH1B1*, *ALDH2*, *CAT*, *CYBA*, *CYP1A1*, *CYP2E1*, *GCLM*, *GLUL*, *GPX*, *GPX2*, *GSS*, *GST*, *GSTM1*, *GSTM5*, *HMOX1*, *IL1B*, *MAO*-A, *MDA*, *NOS*, *NOX*, *NOX2*, *NRF2*, *PTGS2*, *SOD*, *SRXN1*, *TXN*, *TXN1*, and *XDH*. After fitting the EWAS model, adjusted for sex, age, BMI, diabetes, smoking, batch effect, and leukocyte cell counts, we obtained the beta estimates and the *p*-values of the methylation sites on the selected genes. For this candidate gene analysis, the statistical significance was set at the nominal level.

Likewise, we carried out EWAS for the alcohol consumption biomarkers (GGT and the epigenomic score). Other separate regression models, adjusted for the same covariates (sex, age, BMI, diabetes, batch effect, and leukocyte cell counts), were fitted for ln GGT and the epigenomic biomarker. Adjusted *p*-values and correlation coefficients were presented. Likewise, Manhattan plots were displayed.

Gene set enrichment analysis of the differentially methylated CpG sites [128,129] was undertaken for biological and functional interpretation using the Partek Genomics Suite (version 7.20.0831; St. Louis, MO, USA) and Partek Pathway (version 7.0) [72]. Kyoto Encyclopedia of Genes and Genomes (KEGG) pathway analyses and Gene Ontology (GO) enrichment [129,130] were carried out for the top differentially methylated CpG sites obtained in the EWAS for the epigenomic biomarker that passed the false discovery rate (FDR) cut-off. Enrichment scores were computed, and Bonferroni-corrected *p*-values were used to identify which KEGG pathways and GO terms were significantly enriched after correction for multiple tests.

Moreover, we explored the potential modularizing effect of adherence to the Mediterranean diet on the methylation sites associated with the epigenomic biomarker for alcohol consumption. We conducted two additional EWAS for this biomarker (multivariable adjusted), stratified depending on the levels of adherence to the MedDiet (low and high). The corresponding Miami plots were presented.

On the other hand, using the normalized DNAm beta matrix previously obtained, we computed pre-selected biomarkers of aging as previously reported [92]. The selected aging biomarkers were the following: DNAm telomere length, GrimAge, PhenoAge, and the next-generation CausalityAgeYing [88,89,90,91]. For cohort 1 and cohort 2, we estimated the first three metrics of aging using the Horvath epigenetic age calculator [123]. In cohort 1, we additionally calculated the CausalityAgeYing biomarker using the Biolearn platform [124]. The age-adjusted estimate of DNAm telomere length (referred to as DNAmTLadjAge) was derived online by regressing DNAm telomere length on age and obtaining the raw residuals. Similarly, we obtained GrimAgeAccel and PhenoAgeAccel as the residuals resulting from regressing the GrimAge and PhenoAge. For additional comparisons, biomarkers of aging were Z-transformed. Using general linear models adjusted for covariates, we computed the associations between self-reported alcohol consumption (expressed as a categorical variable with 4 categories to capture non-linear effects and as a continuous variable) and the 4 biomarkers of aging in cohort 1. In cohort 2 we analyzed associations with the 3 biomarkers of aging. Regression models were adjusted for sex, age, diabetes, BMI, and smoking (model 1) and additionally adjusted for physical activity, medications, and educational level (model 2). Likewise, we analyzed the association between the selected biomarkers of alcohol consumption (GGT and the epigenomic score) and the aforementioned biomarkers of aging using multivariable-adjusted models, including model 1 and model 2.

Finally, we explored the interaction between self-reported alcohol consumption and Mediterranean diet adherence levels (low and high from the MEDAS-16 scale after removing the wine item in the MEDAS-17) and analyzed the statistical significance of the alcohol–Mediterranean diet interaction term on the aging biomarkers. Figure S1 shows the overview of the main characteristics, the data obtained, and the analyses conducted in the main cohort and in the replication cohort.

## 3. Results

### 3.1. Characteristics of Participants in the Primary Cohort

The demographic, anthropometric, clinical, biochemical, and lifestyle characteristics of the 414 individuals analyzed in cohort 1 are displayed in Table 1. All subjects were participants in the PREDIMED-Plus-Valencia study, and baseline measurements were performed on all parameters. Biological samples for biochemical and methylation analysis were collected on the same day. Participants had metabolic syndrome and the mean age was 65.08 ± 0.24 years. The mean of self-reported alcohol consumption for the entire population was 8.16 ± 0.60 g/d, with statistically significant differences per sex (*p* < 0.001), with men consuming more alcohol than women. Beer consumption (g/d) was greater than wine and spirits among the various types of alcoholic beverages.

**Figure 1:**
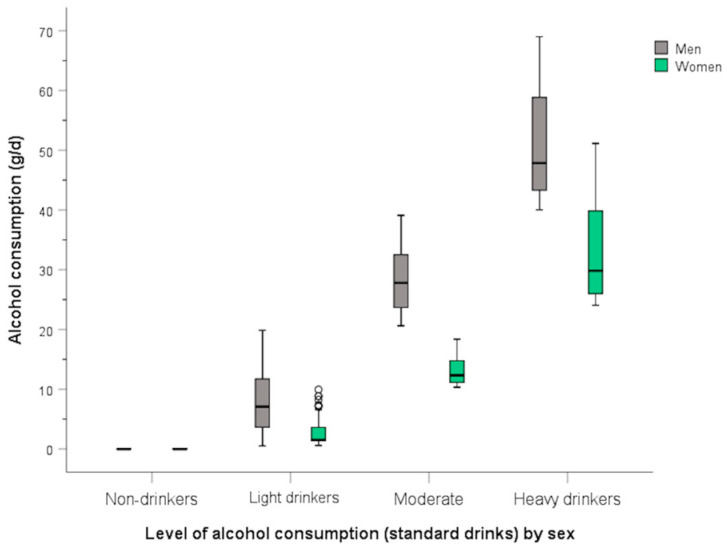
Box plot of the levels of alcohol consumption according to standard drink categories in men and women: non-drinkers (alcohol = 0); light drinkers (<20 g/d in men and <10 g/d in women); moderate drinkers (20 to 40 g/d in men and 10 to 20 g/d in women); and heavy drinkers (≥40 g/d in men and ≥20 g/d in women), (n = 100, 245, 47, and 22, respectively).

Subjects were further classified in four categories of self-reported alcohol consumption according to the cut-off points previously published by Lui et al. [63] (Figure S2). However, considering that this was a population of mostly-low-to moderate alcohol users, the number of individuals in the highest categories was very limited. Additionally, alcohol consumption was categorized using another criterion [111], with narrower thresholds based on the alcohol content of standard drinks in this country (Figure 1).

### 3.2. Characteristics of the Methylation-Based Biomarker of Alcohol Consumption and Association with Alcohol Intake in Cohort 1

Figure S3a illustrates the frequency distribution of the computed epigenomic biomarker for alcohol consumption, derived from methylation data at 450 CpGs and the corresponding weights [77]. Figure S3b depicts the excellent correlation (r^2^ = 1) observed when calculating this score across two distinct platforms. Subsequently, we examined the association between plasma conventional biomarkers of alcohol consumption (ALT, AST, and GGT) as well as the epigenomic score and self-reported alcohol consumption (in g/d, as square-root transformed) (Table 2).

We observed that ALT and AST were not significantly associated with alcohol consumption, whereas plasma GGT levels showed a significant association (*p* < 0.001 even after multivariable adjustment for potential confounders). Likewise, the epigenomic score was strongly associated with self-reported alcohol consumption, even after adjustment for sex, age, BMI, diabetes, smoking, physical activity, medications, educational level, and adherence to the Mediterranean diet (*p* < 0.001).

Next, we analyzed the association between the statistically significant biomarkers in Table 2 and the four categories of self-reported alcohol intake based on standard drinks. Figure S4a shows the box plots illustrating the association with plasma GGT, while Figure S4b displays the boxplot for the epigenomic biomarker. Statistically significant associations with self-reported alcohol consumption (*p* < 0.001) were identified for both biomarkers.

**Figure 2:**
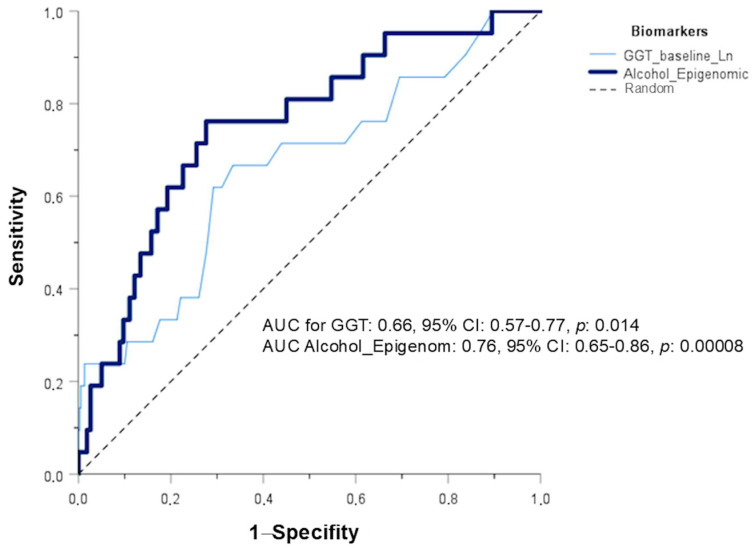
Receiver operating characteristics (ROC) curves for the two selected biomarkers for alcohol consumption (GGT plasma levels and the epigenomic biomarker) in cohort 1 for discriminating heavy drinkers (≥40 g/d in men and ≥20 g/d in women) versus the other groups. The area under the curve (AUC), 95% confidence intervals (CI), and the corresponding *p*-values are presented.

We obtained the ROC curves for each biomarker (plasma GGT levels and the epigenomic biomarker) predicting alcohol intake. First, we tested the performance for high alcohol consumption. Figure 2 shows the ROC curves for plasma GGT and the epigenomic biomarkers for predicting heavy drinkers in cohort 1. The epigenomic biomarker performed better than GGT, producing a higher AUC (0.76; 95% CI: 0.65–0.86; *p* = 0.00008).

However, when comparing drinkers versus non-drinkers, the performance of the epigenomic biomarker was worse (AUC: 0.51, 95%CI: 0.44–0.57, *p* = 0.819), potentially suggesting that some current non-drinkers were former drinkers or that the methylation effect is higher at higher consumption. The predictive performance for current drinkers versus non-drinkers was significant for the GGT biomarker (*p* < 0.001).

**Figure 3:**
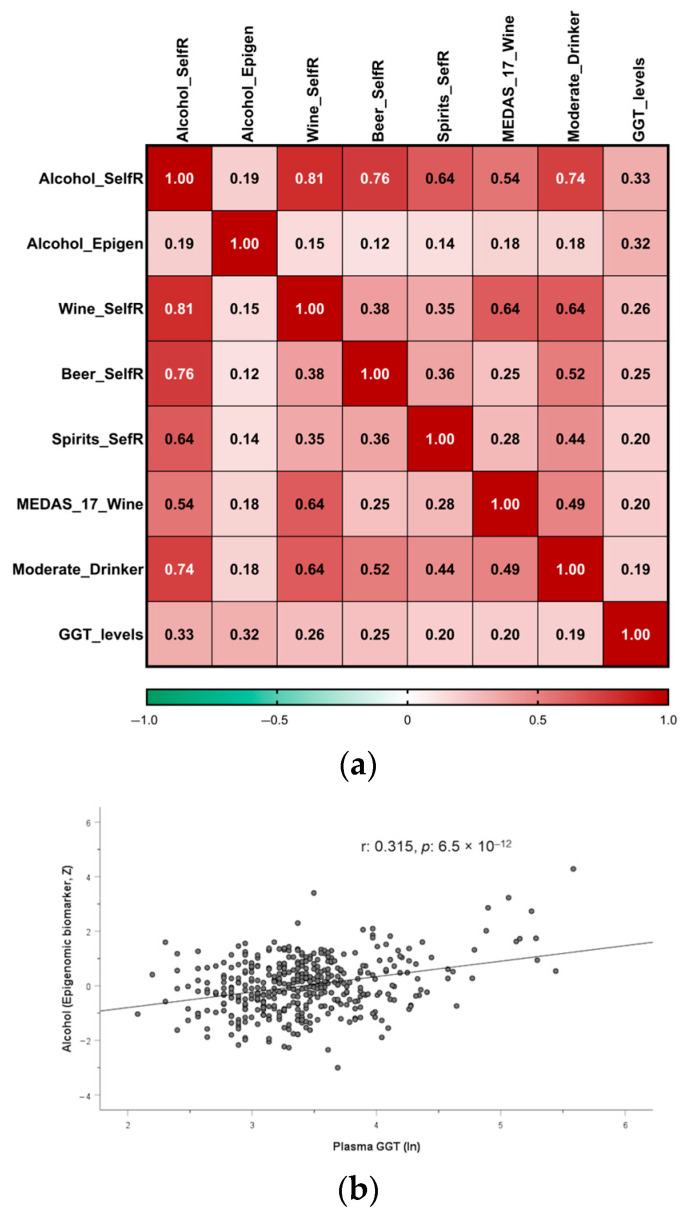
(**a**) Heatmap for the correlation (Pearson) among self-reported alcohol consumption (total and alcoholic beverages) and biomarkers of alcohol intake (epigenomic score and plasma GGT) in PREDIMED-Plus-Valencia participants (n = 414). Color intensity is proportional to Pearson correlation coefficients (see color bar for details: warm red colors represent positive correlations and cool green colors denote negative correlations). Annotations within squares indicate exact correlation (Pearson) coefficient between each pair of variables. Pearson coefficients > 0.09 are statistically significant (*p* < 0.01); (**b**) scatter plot and regression line for the association between biomarkers of alcohol consumption.

We also tested the association between the epigenomic biomarker and self-reported alcohol consumption as continuous variables. Figure 3a displays the Pearson correlation coefficients (including the point-biserial correlation coefficient for dichotomous variables) among self-reported alcohol consumption (g/d), the epigenomic biomarker of alcohol intake, GGT levels, the three types of alcoholic beverages, the moderate drinking, and the dichotomous classification of participants based on their responses to the MEDAS-17 score regarding wine intake. We identified statistically significant correlations (*p* < 0.05) across all examined variables, with a stronger association observed between the epigenomic biomarker score and the levels of GGT (r = 0.32; *p* = 6.5 × 10^−12^). The association between the epigenomic score and self-reported alcohol consumption was statistically significant but lower (r = 0.19; *p* = 8 × 10^−5^). Figure 3b shows the scatter plot and the regression line for the correlation between the epigenomic biomarker and plasma GGT levels in cohort 1.

Figure S5 shows the scatter plots and the regression lines for the relationships between (a) the GGT levels and the self-reported alcohol intake in drinkers and (b) the epigenomic score and the self-reported alcohol intake (in drinkers).

### 3.3. Associations Between the Methylation-Based Biomarker of Alcohol Consumption and Alcohol Intake in the Replication Cohort

The basic characteristics of the replication cohort (n = 150), consisting of men and women of comparable age, from the same geographic area as those in cohort 1, and at high cardiovascular risk, are shown in Table 3.

GGT levels for these participants were not available. Self-reported alcohol consumption was slightly lower in this cohort, and the statistically significant differences indicating higher consumption (*p* < 0.001) among men compared to women were maintained. In this cohort, subjects were also classified in four categories of self-reported consumption based on standard drinks; however, only one man was classified as a heavy drinker and was grouped with the moderate drinkers. Therefore, we estimated the ROC curve for the epigenomic biomarker in this replication cohort to distinguish moderate drinkers from other drinkers, yielding statistical significance (AUC: 0.70; 95% CI: 0.59–0.80; *p* = 0.001) and indicating similar results.

**Figure 4:**
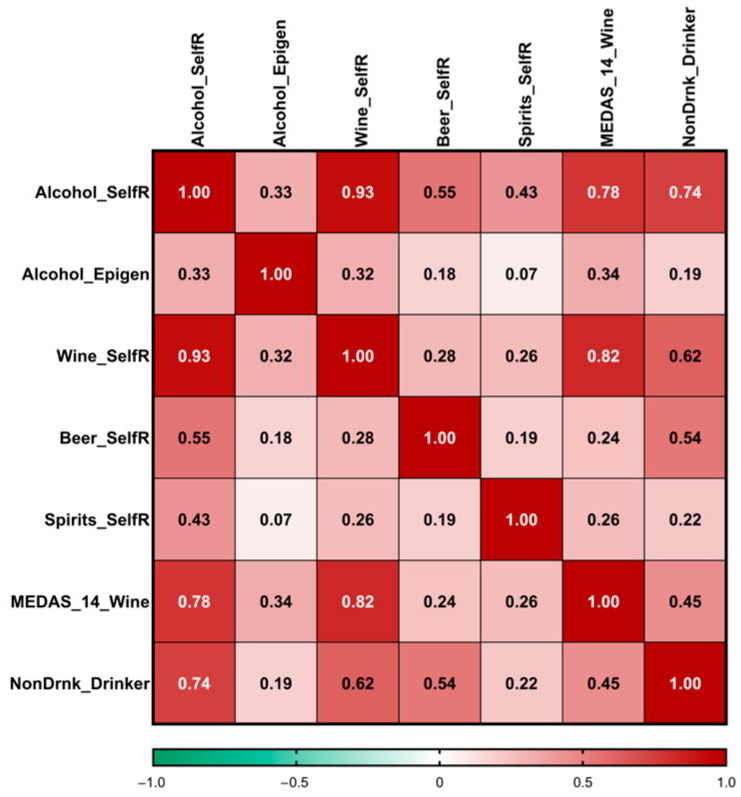
Heatmap for the correlation (Pearson) between self-reported alcohol consumption (total and alcoholic beverages) and the epigenomic biomarker of intake in the replication cohort. Color intensity is proportional to Pearson correlation coefficients (see color bar for details: warm red colors represent positive correlations, and cool green colors denote negative correlations). Annotations within squares indicate exact correlation (Pearson) coefficient between each pair of variables. Pearson coefficients > 0.17 are statistically significant (*p* < 0.01).

Figure 4 displays the Pearson correlation coefficients and the point-biserial correlation coefficient among self-reported alcohol consumption (g/d), the epigenomic biomarker of alcohol intake, the types of alcoholic beverages, non-drinker/drinker status, and the dichotomous classification of participants based on their responses to the MEDAS-14 score concerning wine intake.

We observed a statistically significant correlation between the epigenomic biomarker of alcohol consumption and self-reported alcohol intake (r = 0.33; *p* = 0.00004), replicating the direct association previously identified in the main cohort (cohort 1). Likewise, wine was the type of alcoholic beverage most significantly associated with the epigenomic biomarker.

### 3.4. EWAS for Self-Reported Alcohol Consumption in the Main Cohort

To investigate the effect of alcohol consumption on epigenome-wide methylation in this Mediterranean population, we first analyzed the association of self-reported alcohol intake in cohort 1.

**Figure 5:**
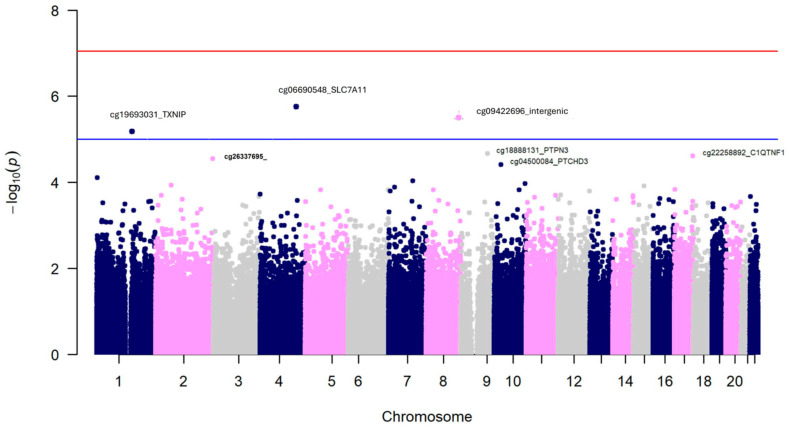
Manhattan plot of the epigenome-wide association for self-reported alcohol consumption (as continuous in g/d and square-root transformed). The regression model was adjusted for sex, age, diabetes, BMI, batch effect, smoking, and leukocyte cell types. The red line represents the EWAS significance level *p* = 9 × 10^−8^. The blue line represents *p* = 1 × 10^−5^. The GRCh37/hg19 assembly is used.

Figure 5 presents the Manhattan plot for the EWAS of self-reported alcohol consumption as a continuous variable (square-root transformed) after adjustment for covariates.

Three CpGs surpassed the statistical threshold for suggestive statistical significance; however, no findings were observed at the EWAS level. This may be explained by a limited sample size or a classification bias inherent in the self-reported consumption data. Interestingly, we detected the cg06690548 as the most statistically significant (*p* = 1.85 × 10^−6^) methylation site, annotated to the *SLC7A11* gene (a cysteine/glutamate transporter) (Table 4). It has been involved in the protection against oxidative damage in response to alcohol-induced oxidative stress [57].

This finding replicates previous research across diverse populations. We also observed a negative correlation between alcohol consumption and methylation at this site (r = −0.24, *p* < 0.001), consistent with prior findings [57] linking the cg06690548-*SLC7A11* hypomethylation to increased alcohol consumption. The second most significant CpG was located in an intergenic region on chromosome 8 and was also hypomethylated with higher alcohol consumption. Similarly, we observed hypomethylation associated with increased self-reported alcohol consumption in the cg19693031 site annotated to the *TXNIP* (Thioredoxin-interacting protein) gene, which encodes a protein that directly interacts with a relevant antioxidant protein thioredoxin [131].

Furthermore, we examined the differential methylation of selected genes associated with oxidative stress, as detailed in the Methods section. Table 5 presents the 14 CpGs with the most significant associations at *p* < 0.05.

We observed hypomethylation (r = −0.142, *p* < 0.05) of cg050100179 in the *CYP1A1* (Cytochrome P450, family 1, subfamily A, polypeptide 1) gene, associated with higher alcohol intake, and hypermethylation of multiple CpGs within the CYP2E1 (Cytochrome P450 Family 2 Subfamily E Member 1) gene (i.e., cg19837601; r = 0.128; *p* < 0.05). Additionally, *ADH5* (Alcohol Dehydrogenase 5 (Class III)), *ALDH2* (Aldehyde Dehydrogenase 2 Family Member), and *ALDH1B1* (Aldehyde Dehydrogenase 1 Family Member B1) exhibited statistically significant differential methylation associated with self-reported alcohol consumption.

### 3.5. EWAS for Plasma Concentrations of GGT

**Figure 6:**
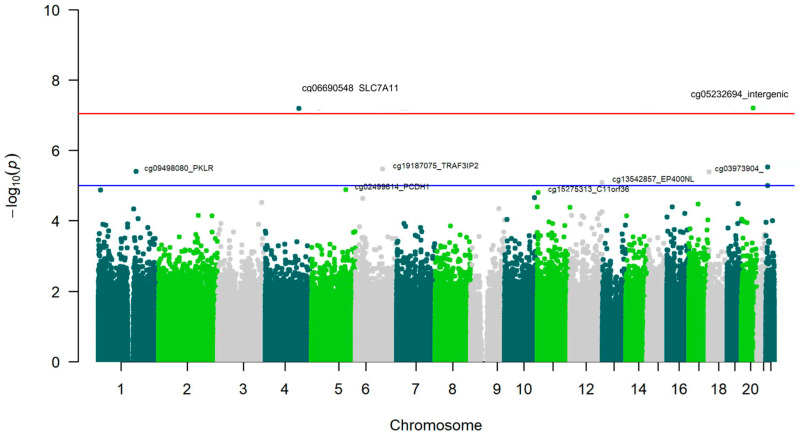
Manhattan plot of the epigenome-wide association for plasma gamma-glutamyl transferase (GGT) levels (multivariable adjusted). GGT values (g/d) were transformed using the natural logarithm. The regression model was adjusted for sex, age, diabetes, BMI, batch effect, smoking and leukocyte cell types. The red line represents the EWAS significance level *p* = 9 × 10^−8^. The blue line represents *p* = 1 × 10^−5^.

Subsequently, we conducted an EWAS for plasma GGT (ln) as a biomarker of alcohol intake. We observed (Figure 6 and Table 6) that, for this more objective biomarker, the cg06690548 in the *SLC7A11* locus achieved statistical significance at the EWAS level (*p* = 3.03 × 10^−8^).

Elevated plasma GGT levels were correlated with hypomethylation of this site, consistent with the findings related to self-reported alcohol intake. The other differentially methylated CpGs identified at the EWAS level or at the suggestive level of significance, including the *GGT1* (Gamma-Glutamyltransferase 1) gene, among others, were consistent with prior research.

### 3.6. EWAS for the Epigenomic Biomarker

**Figure 7:**
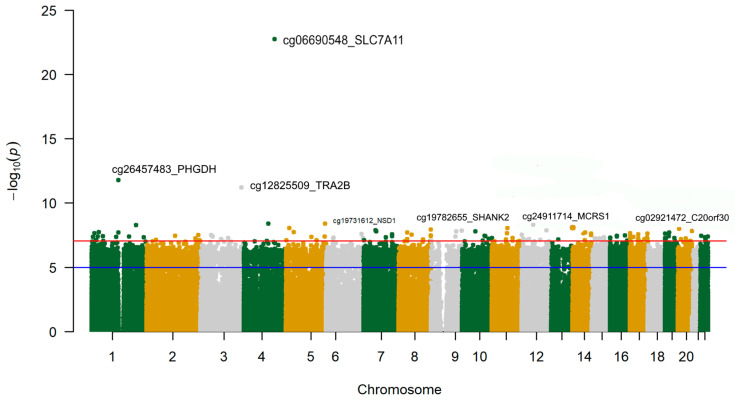
Manhattan plot of the epigenome-wide association for epigenomic biomarker of alcohol consumption (based on the 450-CpGs score) (multivariable adjusted). The regression model was adjusted for sex, age, diabetes, BMI, batch effect, smoking, and leukocyte cell-types. The red line represents the EWAS significance level *p* = 9 × 10^−8^. The blue line represents *p* = 1 × 10^−5^. The GRCh37/hg19 assembly has been used.

Similarly, we conducted an EWAS for the epigenomic score of alcohol consumption (Figure 7 and Table 7).

Although this score was derived from methylation levels at specific CpGs, the purpose of conducting this EWAS was to better understand the significance of the computed score within this population and to elucidate the underlying factors contributing to the scores. This EWAS should be interpreted as biological annotation rather than independent discovery. As expected, the leading site in the EWAS was the cg06690548-*SLC7A11*, consistent with the score and the other EWAS. We also detected hypomethylation of the site. The following most significant sites were in the *PHGDH* (Phosphoglycerate Dehydrogenase) gene and in the *TRA2B* (Transformer-2 Protein Homolog Beta) gene, both hypomethylated and involved in oxidative stress.

#### 3.6.1. Enrichment Analysis (KEGG and GO Pathways and Functions)

To further elucidate the score information, KEGG pathway analyses and GO functional enrichment were performed on the top differentially methylated CpG sites identified in the corresponding EWAS for the epigenomic biomarker that met the FDR threshold. In this EWAS, Table 8 presents the 25 most significant pathway names, along with their associated enrichment *p*-values and Bonferroni-adjusted enrichment *p*-values. A total of 128 sites passed the EWAS threshold of significance (*p* < 9 × 10^−8^), and 19,373 sites passed the false discovery rate (FDR) level.

We identified multiple KEGG pathways (n = 35) that met the criteria for statistical significance after Bonferroni correction. Among these highly enriched pathways, we identified five explicitly associated with the cancer phenotype; additionally, pathways related to neurodegeneration and aging were also found to be relevant.

The most statistically significant finding was the “Rap1 signaling pathway,” associated with oxidative stress and telomere length, followed by “pathways in cancer.” Table S2 presents the first 30 GO enrichment functions, showing their category (biological process, cellular component, or molecular function), enrichment score, raw enrichment *p*-value, and Bonferroni-adjusted enrichment *p*-value. The majority of the findings that reached statistical significance pertained to biological processes. The notable findings were “Organelle” and “protein binding,” both associated with highly statistically significant *p*-values. These results were in agreement with previous findings in other populations.

#### 3.6.2. Exploratory Analysis of EWAS Modulation by Mediterranean Diet Adherence

**Figure 8:**
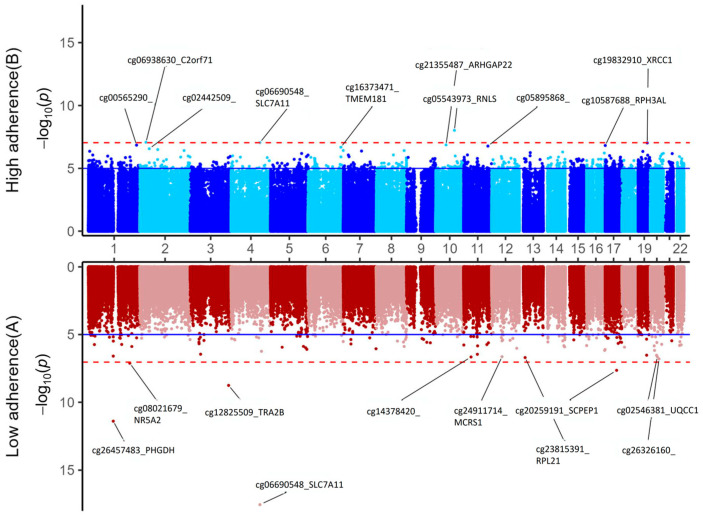
Miami plot for the epigenome-wide methylation analysis (adjusted for all the covariates indicated in Figure 7) on the epigenomic biomarker of alcohol consumption depending on the adherence to the Mediterranean diet using the MEDAS-17 scale: high adherence (9–17 points) (above *x*-axis) and low adherence (0–8 points) (below *x*-axis). For the high adherence level, the 10 most significant CpGs are highlighted. The top 10 most significant CpGs for the low adherence level are also highlighted. The red line represents the EWAS significance level *p* = 9 × 10^−8^. The blue line represents *p* = 1 × 10^−5^. The GRCh37/hg19 assembly has been used.

To explore the potential effect of an antioxidant-rich context on the methylation effects of alcohol consumption on the epigenome, we performed a stratified EWAS of the epigenomic score for alcohol consumption in cohort 1 (multivariable-adjusted model), stratifying by high versus low adherence to the Mediterranean diet. Figure 8 presents the Miami plot for both stratified EWAS. The results suggested an apparent difference in methylation profiles depending on the level of the adherence.

For example, regarding cg06690548-*SLC7A11*, this site exhibited high statistical significance when adherence to the Mediterranean diet was low (the hit at *p* = 2.9 × 10^−18^). However, when adherence to the Mediterranean diet was high, its differential methylation was less pronounced (*p* = 8.8 × 10^−8^), suggesting a potential modulating effect of the antioxidants present in a high level of adherence to the Mediterranean diet in the methylation profile of CpGs linked to alcohol consumption. Thus, the 5 top-ranked genes in the strata of low adherence to the Mediterranean diet by statistical significance were *SLC7A11*, *PHGDH*, *TRA2B*, *SCPEP1* (Serine Carboxypeptidase 1), and *NR5A2* (Nuclear Receptor Subfamily 5 Group A Member 2); whereas in the strata of high adherence to the Mediterranean diet, the top 5 genes were *RNLS* (Renalase), *C2orf71* (Chromosome 2 Open Reading Frame 71), *SLC7A11*, *XRCC1* (X-ray Repair Cross-Complementing 1), and *ARHGAP22* (Rho GTPase Activating Protein 22). Nevertheless, this is only an exploratory analysis, and the results are hypothesis generating.

### 3.7. Associations Between Self-Reported Alcohol Intake and Biomarkers of Alcohol Consumption with Selected Biomarkers of Aging

**Figure 9:**
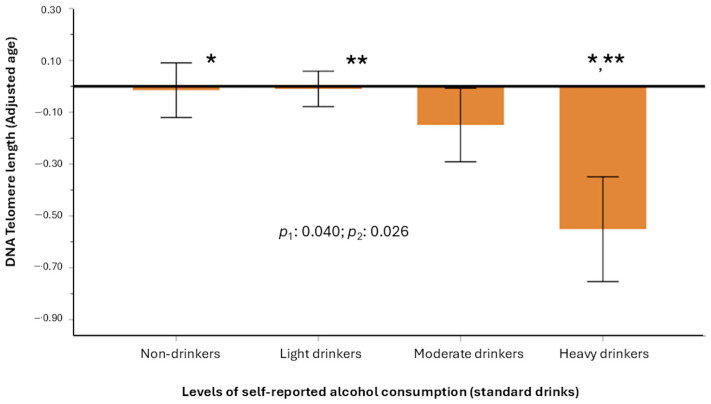
DNAm telomere length (adjusted for age and z-transformed) depending on the self-reported alcohol intake (4 categories). Regression models were adjusted for: (1) sex, age, diabetes, BMI and smoking; (2) additionally adjusted for physical activity, medication and education. *p*-values for trend in the multivariable models. *: Statistically significant differences with heavy drinkers; **: Statistically significant differences with heavy drinkers. Error bars: SE of means (standard errors).

We first examined the association between self-reported alcohol consumption and biomarkers of aging in cohort 1. Figure 9 illustrates the mean telomere lengths (adjusted for age and Z-transformed) in cohort 1 according to the four categories of self-reported alcohol consumption based on standard drinks.

In the general model, we identified statistically significant associations between increased self-reported alcohol consumption (*p* for trend) and reduced telomere length, both in the model adjusted for sex, age, diabetes, BMI, and smoking (model 1) (*p* = 0.040) and in the model additionally adjusted for physical activity, medications, and educational level (model 2) (*p* = 0.026) when analyzing categories. Heavy drinkers exhibit a significant reduction in telomere length compared to non-drinkers and with light drinkers. However, using the same models, we did not observe any statistically significant association between self-reported alcohol consumption and GrimAgeAccel (Figure S7a) or PhenoAgeAccel (Figure S7b). Similarly, no association was observed between self-reported alcohol intake and the CausAgeYing aging biomarker (*p* > 0.05).

However, when we tested the association between the epigenomic biomarker of alcohol consumption and the four biomarkers of aging (as continuous variable), we obtained highly statistically significant association with all the biomarkers in cohort 1 (Table 9). Figure S8 shows the scatter plots for (a) DNAm telomere length, and (b) GrimAgeAccel. Table 9 shows the association of the epigenomic biomarkers of alcohol consumption with the four biomarkers of aging adjusted for several confounders.

Associations remained statistically significant even after adjustment for the full model. The association was inverse for telomere length and direct for GrimAgeAccel, PhenoAgeAccel, and the CausAgeYing clock. For all the clocks, the association remained statistically significant even with fully multivariable adjustment (all *p* < 0.001).

When testing the associations between the epigenomic biomarker of alcohol intake and biomarkers of aging in cohort 2, despite the limited sample size, we obtained statistically significant associations. Figure S9 shows the corresponding scatter plots for (a) DNAm telomere length and (b) GrimAgeAccel. We observed statistically significant associations for both. Moreover, after multivariable adjustment (model 3) of Table 9, we obtained a statistically inverse association between the epigenomic score for alcohol consumption and DNAm telomere length (beta: −0.044; *p* = 0.027) and a direct association between the epigenomic score and the GrimAgeAccel (*p* < 0.001), replicating the findings observed in cohort 1. However, no significant association was detected for PhenoAgeAccel (*p* = 0.068).

Moreover, in cohort 1, the plasma biomarker of GGT levels was also significantly associated with the aging biomarkers in multivariable models. For telomere length, it was inversely associated (*p* = 0.030), but for GrimAgeAccel, PhenoAgeAccel, and CausAgeYing, we obtained statistically significant direct associations (*p* = 0.001, *p* = 0.013, and *p* = 0.007, respectively). No GGT data was available for cohort 2.

Finally, we explored the potential interaction of the alcohol intake and the level of adherence to the Mediterranean diet on telomere length. Low and high levels of adherence (0–8; and 9 and more, respectively) were used in the score without including the point for wine intake. Figure S10 shows DNA telomere length metrics depending on alcohol consumption (four categories) and the level of adherence to the Mediterranean diet. The interaction term was not statistically significant (*p* = 0.134) when analyzing the four categories of alcohol intake, but, considering the limitations of the sample size, we grouped the four categories of alcohol intake into two groups: non-drinkers + light drinkers versus moderate and high drinkers. When we tested the interaction term using these categories of alcohol consumption, it reached statistical significance (*p* < 0.05) (Figure S11), suggesting that a higher adherence to the Mediterranean diet may modulate the effects of alcohol consumption on shorter telomere length. Next, we explored these interactions for the other biomarkers, but we did not obtain statistically significant results. Considering that this is an exploratory analysis undertaken for the first time, additional more focused studies with larger sample sizes are needed.

## 4. Discussion

This study evaluated the performance of a methylation-based score as a biomarker of alcohol consumption within an older population characterized by low-to-moderate self-reported alcohol intake. This is the first time this association has been investigated in a Mediterranean population, and the findings are valuable for assessing the external validity of this methylation-based score in populations beyond the original cohort [77]. It is well known that self-reported consumption of alcohol is a complex phenotype susceptible to recall bias, to social desirability, and to other limitations [73,132]. Therefore, in light of the impact of alcohol consumption on the epigenome [67,68,133], there is considerable interest in the development and validation of novel epigenetic biomarkers based on DNA methylation [134] that facilitate a more objective assessment of alcohol consumption. Although it is an emerging area of research, a few DNA-based methylation scores for alcohol consumption have already been proposed [63,77,135,136], with the most commonly utilized being those developed by Liu et al. [63] and by McCartney et al. [77]. Liu et al. [63] were among the initial researchers to investigate such scores. They devised four blood DNA methylation-based scores as potential biomarkers of alcohol intake within the Heart and Aging Research in Genomic Epidemiology Consortium plus (CHARGE+) Consortium cohorts. Among the four scores consisting of 5 CpG, 23 CpG, 78 CpG, and 144 CpG sites, the score with the highest number of CpG sites (144-CpG) demonstrated the best performance. Subsequent studies have employed this 144-CpG score as a biomarker for alcohol consumption [69,78,79,137,138,139,140] and its association with several phenotypes, yielding mixed results. A criticism of this score concerns its propensity for overfitting [141] and the fact that it was trained to discriminate heavy drinkers [63].

Consequently, we did not choose the 144-CpG score, proposed by Lin et al. [63], for validation in our Mediterranean cohort. Instead, we selected the methylation-based score trained for habitual alcohol consumption (in a typical week) developed by McCartney et al. [77]. It consists of a 450-CpG methylation-based score, with the discovery sample drawn from participants in the “Generation Scotland: The Scottish Family Health Study,” and the validation cohort from the “The Lothian Birth Cohort 1936.” In the original paper, moderate results were obtained in comparison with tobacco smoking, but this score for alcohol intake strongly predicted mortality risk in comparison with other alcohol metrics (self-reported or genetic) [77]. The 450-CpG score has been used in several studies as a biomarker of alcohol consumption [78,79,138,142], but there have been no prior studies in an elderly Mediterranean population characterized by low-to-moderate alcohol consumption and a more Mediterranean drinking pattern. The advantage of the 450-CpG score compared to other methylation-based scores is that its calculation is automatically implemented on a Horvath epigenetic age online calculator [123]. It can also be calculated directly through the Biolearn platform [124]. Therefore, it can be obtained in a highly standardized manner across many populations using these platforms for biomarkers of aging. Therefore, more results from published articles are expected soon, making it of great comparative interest to obtain data on its validity in an elderly Mediterranean population.

We have tested the 450-CpG epigenomic biomarker in the main cohort (cohort 1), as well as in a replication cohort (cohort 2) from the same geographical area and of similar age, with similar lifestyle and health characteristics. When we tested the association between the epigenomic biomarker of alcohol and the self-reported total alcohol consumption in cohort 1, the association was statistically significant even after adjustment of the main potential confounders (*p* < 0.001), both in the primary cohort and in the replication cohort. This contributes to increasing the validity of the biomarker in the Mediterranean population. However, the magnitude of the association was relatively small (r = 0.2 in cohort 1 and r = 0.3 in cohort 2; *p* < 0.001). Although these figures are in the range of the obtained coefficients in other populations (ranging from r = 0.1–0.4) [77,78,79,138,142], the variability explained was not very high. Therefore, we believe there is potential to improve this methylation-based score to optimize its performance as a biomarker of alcohol consumption in the Mediterranean population.

When analyzing the correlation between the self-reported alcohol consumption and the alcohol intake detected by the epigenomic biomarker, one must not only consider the biases [73,76,132] in reporting alcohol intake (recall bias, social desirability, serving sizes, etc.)—which mean that self-reported alcohol consumption is not actually the alcohol consumed—but also take into account possible differences in the longitudinal alcohol consumption [60,137]. Typically, self-reported alcohol consumption refers to a specific time frame (primarily one week, one month, or one year, in our questionnaire), whereas epigenomic alcohol consumption may reflect an epigenetic footprint left by alcohol consumption over many years that does not correspond to alcohol intake at the time of the questionnaire [140]; for example, people who report being non-drinkers, but who drank in the past [60,137,140]. Therefore, alcohol consumption questionnaires should also be improved to include questions about temporary changes that may be relevant for understanding the differences between measures [132].

In our study, we did not have data on prior alcohol consumption, which is a limitation when interpreting the results of the epigenomic biomarker of alcohol intake. In such cases, it is very useful to have longitudinal data to prospectively analyze changes in consumption. In this study, we did have alcohol consumption data for each year, but we lacked methylation data from the follow-up, so we could not analyze this dynamic characteristic. We are planning it as future research to improve this biomarker.

Most studies examining the influence of alcohol consumption on DNA methylation have been cross-sectional [56,58,59,63,65,77,88,89,143]. However, there are also some longitudinal studies [60,64,95,137] that have been able to analyze how methylation at specific CpG sites changes following alterations in alcohol consumption. As with tobacco use [71,72], it appears that there are some CpG sites where the imprint of alcohol consumption at a given time is more permanent, while at other CpG sites methylation is more dynamic and better reflects recent alcohol intake [55,68]. Thus, Dugué et al. [60] have provided data on the so-called “reversibility coefficient” (assessing for former drinkers the degree to return to the average methylation levels of never drinkers), integrating longitudinal methylation data of participants with important changes in alcohol intake. They found great variability; for example, for the cg11376147, the reversibility coefficient was reported to be small (7%), whereas for the cg16246545, the reversibility coefficient was very high (100%). In agreement with these observations, the methylation-based biomarker of alcohol intake may be a long-term indicator of alcohol consumption instead of a short-term biomarker of intake.

In our population, we have observed a higher correlation coefficient between the epigenomic biomarker and the conventional plasma GGT biomarker [74] than between the self-reported alcohol intake and the epigenomic biomarker, possibly suggesting that long-term alcohol intake is more correlated with the epigenomic biomarker than short-term intake. A more focused analysis, including a larger sample size and additional metabolomics biomarkers [75,76], is needed. Similarly, other metabolomics biomarkers have been more thoroughly characterized within the time window, exhibiting the strongest correlation with self-reported intake [75]. Ethyl glucuronide can be detected in urine for up to five days, whereas phosphatidylethanol in blood remains detectable for two to three weeks or longer [75]. In our study, we did not have measurements of these metabolomics biomarkers, but since an EWAS has been published analyzing the differentially methylated loci associated with phosphatidylethanol [144], it would be interesting to examine the association between the epigenomic biomarker of alcohol intake and the published epigenetic biomarker of phosphatidylethanol to see whether it reflects a stronger or weaker relationship with the timing of alcohol consumption in this Mediterranean population.

Although self-reported alcohol consumption as a continuous variable does not show a strong association with the epigenomic biomarker of alcohol intake, we have found that the 450-CpG epigenomic biomarker has a very strong predictive ability to distinguish heavy drinkers from the others. In the original study [63], the AUC for discriminating heavy drinkers was 0.73 (95% CI = 0.69–0.78), whereas in our study it was slightly higher (AUC = 0.76; 95% CI = 0.65–0.86). This would support the score’s stronger association with long-term heavy alcohol consumption.

Regarding the EWAS results, as far as we know, this is the first time that an EWAS on alcohol consumption has been undertaken in the Spanish Mediterranean population. In our main cohort, we found strong consistency in the hypomethylation of the *SLC7A11* gene across the three EWAS conducted (self-reported alcohol consumption, GGT biomarker, and epigenomic biomarker). Although the limited sample size for the EWAS on self-reported alcohol consumption, coupled with its greater variability, did not allow us to achieve statistical significance at the standard EWAS level for the cg06690548-*SL7CA11* site was the most significant. In the EWAS of the GGT biomarker, the cg06690548-*SL7CA11* was the most statistically significant, reaching the EWAS significance threshold and replicating previous findings showing this site as the hit for GGT plasma levels in an EWAS analysis [145].

Although the results of previous EWAS [54,55,56,57,58,59,60,61,62,63,64,65,66] for alcohol consumption have been heterogeneous in terms of the hits identified, since they depend on the measure of alcohol consumption used, the population type, and its characteristics, there is consensus that alcohol consumption is associated with hypomethylation at the cg066905448 site in the *SLC7A11* gene [57]. This is important because *SLC7A11* encodes the light chain of the cysteine/glutamate antiporter. The imported cysteine is used for the synthesis of glutathione (GSH), a major cellular antioxidant [146]. Alcohol generates ROS, causing oxidative stress and hypomethylation of the cg06690548-*SL7CA11* site. Moreover, it has been reported that hypomethylation is linked to overexpression of the gene [57]. However, more complexity in this process has been published [147].

Similarly, another top hypomethylated site in our EWAS for self-reported alcohol was the cg19693031-*TXNIP*, encoding for a protein relevant in the oxidative stress balance [131]. Thioredoxin is an antioxidant protein that reverses protein cysteine oxidation and facilitates scavenging reactive oxygen species [148]. Moreover, when we examined the methylation of pre-selected genes related to oxidative stress, we observed that several sites in relevant oxidative stress genes reached statistical significance. Thus, we detected a hypomethylated site in the *CYP1A1* gene associated with high self-reported alcohol consumption. The hypomethylation of this gene has been related to increased oxidative stress and DNA damage [149]. Also, we observed hypermethylation of several CpGs in the *CYP2E1* gene associated with higher alcohol consumption. Ethanol metabolism in vivo is primarily mediated by *CYP2E1*. It induces ROS production and results in oxidative stress and DNA damage [150]. In the pathway analysis carried out on the epigenomic biomarker of alcohol intake, we detected the “Rap-1 signaling pathway” as the most statistically significant. It has been reported that Rap-1 ameliorates oxidative stress and has anti-inflammatory actions decreasing ROS [151]. This pathway has also been highly ranked in the EWAS conducted within the E3N cohort [54]. In this EWAS for alcohol consumption enrichment analysis, the “MAPK signaling pathway” and “Pathways in cancer” were also among the top-ranked pathways, consistent with our findings.

Finally, in this Mediterranean population, we observed a consistent, statistically significant inverse association between increased alcohol consumption and a decrease in the telomere length biomarker of aging [91]. This effect has been observed in relation to self-reported alcohol consumption (mainly with a high consumption) with plasma GGT and with the epigenomic biomarker. A strength of our study is the utilization of a DNA methylation-based biomarker for telomere length, previously validated by us within this population [92], rather than relying on traditional measurement methods [47], thereby enhancing the accuracy of the estimates [93]. Many studies have examined the effect of alcohol consumption on telomere length [42,43,44,45,46,48,49,91,93], highlighting the role of oxidative stress in the potential mechanisms [38,152]. In general, it can be stated that higher alcohol consumption is associated with shorter telomere length. However, the study results have been mixed, as the methodology used to measure telomere length, the population characteristics, and the instruments for assessing alcohol consumption and its quantity have varied.

In our study, we found an inverse association with self-reported alcohol consumption, primarily at high levels of intake. One limitation of our study is that alcohol consumption is relatively low and the sample size for the high-consumption group is small. However, the effect is very large, and the results are statistically significant. Likewise, for the plasma GGT biomarker and the epigenomic biomarker of alcohol consumption, we have found statistically significant inverse associations in this Mediterranean population (main cohort). Moreover, we replicated, in cohort 2, the inverse association between the epigenomic biomarker of alcohol consumption and telomere length. A limitation of the replication cohort was the lack of available plasma GGT values, along with the small sample size that prevented us from conducting an EWAS analysis. However, the strength of the replication cohort was recruitment from the same geographical area, having similar general socio-demographic and clinical characteristics, and the use of the same laboratory and questionnaires to derive alcohol consumption and other related variables. Unlike the consistency found between self-reported alcohol consumption and its biomarkers in the association with telomere length, for the other aging biomarkers analyzed (GrimAgeAccel, PhenoAgeAccel, and CausAgeYing) [88,89,90], we did not find significant results for the self-reported alcohol measure, although we did for the alcohol consumption biomarkers—both GGT concentrations and the epigenomic biomarker in cohort 1. For all of them, we found a statistically significant positive association (more significant for the epigenomic biomarker than for the GGT concentrations) even after adjusting for the influence of potential confounders. Likewise, in the replication cohort, we found a strong direct association between the epigenomic biomarker of alcohol consumption and GrimAgeAccel.

The results of other studies have also been diverse, ranging from inverse associations with GrimAge and the EEAA with low-to-moderate alcohol consumption to direct associations and no associations with these or other amounts [56,58,88,89,94,95,96,97,98,99,153]. This discrepancy between the results obtained from self-reported alcohol consumption and epigenomic biomarkers may occur because the epigenomic marker is a better indicator of lifetime intake and more accurately captures aging risk than a single-point measure of consumption reported in a questionnaire. Further longitudinal studies with morbidity and mortality data are needed to better understand these factors. Another aspect that could influence this is a potential overlap among the CpG sites included in the 450-CpG epigenomic score and those used to construct the aging biomarkers. Therefore, we analyzed the overlaps (Table S3). We observed that for telomere length there is no common CpG locus between the 450-CpG score and the biomarker of aging. However, for the other aging biomarkers, there is a slight overlap. The site cg06690548-*SLC7A11* is present in both GrimAge and PhenoAge. Additionally, in GrimAge there are four other methylation sites that overlap (in *ALK*, *KIRREL3*, *PHGHG*, and one intergenic loci). For CausAgeYing, one overlap is only detected in the *CACNG6* gene. More research is needed to better understand the influence of such partial overlap.

Finally, it has been suggested that the dietary context might influence alcohol’s epigenome effects [154]. Since dietary antioxidants may modulate the effects of alcohol intake, we explored the influence of the low adherence to the Mediterranean diet (low in antioxidants) versus high adherence (high in antioxidants) [100,101] on DNA methylation and aging biomarkers. Taking into account the exploratory nature of our findings and the sample size limitations, our results may suggest that a high Mediterranean diet adherence may mitigate some negative effects of alcohol consumption on specific CpGs and aging biomarkers (mainly telomere length). However, it is a hypothesis driving study, and further research is needed to investigate and extend these findings.

An important limitation of this study, apart from the limited sample size and the lack of EWAS results and certain biomarkers in cohort 2, is that it was conducted on subjects aged 55–75 years in the main cohort and aged 55–80 years in the replication cohort. Hence, the findings may not be generalizable to other populations differing in the age range. Also, the analyzed cohorts were enriched in subjects with high cardiovascular risk, and patterns of alcohol consumption may vary among healthy individuals, as could the performance of the epigenomic biomarker. In addition, it is important to consider the limitations of self-reported alcohol consumption based on questionnaires, which are prone to bias. Participants can lie in their self-reports about the amount of alcohol consumed, which may lead to an underestimation that may differ by sex or by other characteristics, underscoring the need for more objective measures of alcohol intake. Nevertheless, this is the first time that the impact of alcohol consumption on the methylome and the performance of the epigenomic biomarker of alcohol intake has been analyzed in this Mediterranean population. Consequently, our results are relevant for future research.

## Figures and Tables

**Table 1:** Demographic, clinical, and lifestyle characteristics of the main cohort according to sex.

	Total (n = 414)	Men (n = 186)	Women (n = 228)	*p*
Age (years)	65.08 (0.24)	63.84 (0.39)	66.09 (0.27)	<0.001
BMI (Kg/m^2^)	32.31 (0.18)	32.16 (0.25)	32.44 (0.25)	0.440
Systolic blood pressure (mmHg)	141.92 (0.91)	143.72 (1.35)	140.45 (1.23)	0.076
Diastolic blood pressure (mmHg)	80.98 (0.49)	82.61 (0.74)	79.64 (0.63)	0.002
Total cholesterol (mg/dL)	195.73 (1.84)	188.11 (2.84)	201.95 (2.34)	<0.001
HDL cholesterol (mg/dL)	51.59 (0.57)	47.34 (0.79)	55.06 (0.72)	<0.001
LDL cholesterol (mg/dL)	124.30 (1.51)	121.51 (2.37)	126.57 (1.94)	0.096
GGT (U/L) ^1^	36.66 (1.50)	43.46 (2.57)	31.06 (1.64)	<0.001
ALT (U/L)	28.59 (0.80)	30.77 (1.18)	26.82 (1.09)	0.014
AST (U/L)	26.39 (0.42)	27.58 (0.66)	25.42 (0.52)	0.010
Triglycerides (mg/dL) ^1^	141.28 (2.95)	139.02 (4.01)	143.13 (4.23)	0.488
Fasting glucose (mg/dL)	113.43 (1.38)	113.67 (2.16)	113.23 (1.79)	0.875
Physical activity (MET.min/wk)	1708 (77)	1940 (132)	1518 (89)	0.007
Mediterranean diet score ^2^	8.04 (0.14)	7.85 (0.20)	8.20 (0.18)	0.210
Total energy intake (Kcal/d)	2400 (31)	2556 (48)	2273 (38)	<0.001
Alcohol intake (g/d) ^3^	8.16 (0.60)	13.73 (1.05)	3.63 (0.49)	<0.001
Wine intake (g/d) ^3^	36.63 (3.25)	60.60 (6.15)	17.08 (2.45)	<0.001
Beer intake (g/d) ^3^	91.48 (9.24)	139.56 (14.01)	52.26 (11.68)	<0.001
Spirits intake (g/d) ^3^	4.29 (0.69)	8.79 (1.44)	0.61 (0.30)	<0.001
Drinkers (%)	75.85	90.86	63.60	<0.001
Diabetes (%)	39.61	40.32	39.04	0.790
Smoking				<0.001
Current (%)	11.59	16.13	7.89	
Former (%)	43.00	66.67	23.68	
Never (%)	45.41	17.20	68.42	
Education				<0.001
Primary (%)	61.59	49.46	71.49	
Secondary (%)	21.01	29.57	14.04	
University (%)	17.39	20.97	14.47	

Values are mean ± SE for continuous variables and % for categorical variables. *p*: *p*-value for differences between men and women. GGT: gamma-glutamyl transferase; ALT: alanine aminotransferase; AST: aspartate aminotransferase; MET: metabolic equivalent. ^1^ GGT and triglyceride values were logarithmically transformed for statistical testing. ^2^ Measured by the MEDAS-17-item questionnaire. ^3^ Alcohol variables were transformed using the square root for statistical testing.

**Table 2:** Association between pre-selected biomarkers of alcohol consumption and self-reported alcohol intake.

Biomarker	Beta ^1^ (SE ^1^)	*p* ^1^	*p* ^2^	*p* ^3^
ALT (U/L)	0.046 (0.032)	0.150	0.152	0.199
AST (U/L)	0.030 (0.062)	0.634	0.625	0.686
GGT (U/L)	0.106 (0.017)	<0.001	<0.001	<0.001
Epigenomic score	0.584 (0.163)	<0.001	<0.001	<0.001

ALT: alanine aminotransferase; AST: aspartate aminotransferase; GGT: gamma-glutamyl transferase. ^1^ Regression model adjusted for sex, age, diabetes, BMI, and smoking; ^2^ model 1 additionally adjusted for physical activity, medications, and educational level; ^3^ model 2 additionally adjusted for adherence to Mediterranean diet. Biomarkers and alcohol consumption were analyzed in original units (as continuous variables) for beta estimates and transformed for statistical testing.

**Table 3:** Demographic, clinical, and lifestyle characteristics of the replication cohort (PREDIMED-Valencia) according to sex.

	Total (n = 150)	Men (n = 65)	Women (n = 85)	*p*
Age (years)	67.59 (0.50)	67.17 (0.81)	67.91 (0.63)	0.466
BMI (Kg/m^2^)	30.42 (0.33)	29.99 (0.45)	30.75 (0.46)	0.251
Fasting glucose (mg/dL)	116.70 (3.29)	122.78 (5.47)	112.00 (3.97)	0.104
Total cholesterol (mg/dL)	211.33 (3.01)	205.45 (4.35)	215.88 (4.09)	0.085
Mediterranean diet score ^1^	8.40 (0.17)	8.42 (0.27)	8.39 (0.23)	0.939
Alcohol intake (g/d)	6.09 (0.76)	10.53 (1.48)	2.68 (0.47)	<0.001
Wine intake (g/d)	41.08 (6.11)	73.36 (12.25)	16.39 (3.58)	<0.001
Beer intake (g/d)	39.89 (6.54)	56.76 (11.51)	26.99 (7.24)	0.024
Spirits intake (g/d)	1.40 (0.50)	2.79 (1.09)	0.33 (0.26)	0.015
Drinkers (%)	62.7	80.0	49.4	<0.001
Current smoker (%)	13.3	20.0	8.2	<0.001
Diabetes (%)	31.3	36.9	27.1	0.197

Values are mean ± SE for continuous variables and % for categorical variables. *p*: *p*-value for differences between men and women. ^1^ Measured by the MEDAS-14-item questionnaire for adherence to the Mediterranean diet. Alcohol variables were transformed using the square root for statistical testing.

**Table 4:** Top 20 differentially methylated CpG sites identified in the EWAS on self-reported alcohol ^1^ consumption ranked by smallest *p*-value after multivariate adjustment.

CpG	Gene Symbol	bp	Chr	CpG Region ^2^	*p*	r
cg06690548	*SLC7A11*	139,162,808	4		1.85 × 10^−6^	−0.2379
cg09422696	*intergenic*	144,626,190	8	S_Shelf	2.42 × 10^−6^	−0.2352
cg19693031	*TXNIP*	145,441,552	1		6.95 × 10^−6^	−0.2246
cg18888131	*PTPN3*	112,172,827	9		2.12 × 10^−5^	0.2127
cg22258892	*C1QTNF1*	77,042,315	17	N_Shore	2.42 × 10^−5^	0.2112
cg26337695	*intergenic*	241,290,620	2	N_Shelf	2.79 × 10^−5^	0.2097
cg04500084	*PTCHD3*	27,703,547	10	S_Shore	3.86 × 10^−5^	−0.2060
cg18502684	*PLCH2*	2,425,952	1		7.75 × 10^−5^	0.1980
cg05845765	*CUX1*	101,880,148	7	N_Shelf	9.07 × 10^−5^	0.1962
cg00135791	*intergenic*	130,275,314	10		1.05 × 10^−4^	−0.1944
cg11016420	*intergenic*	65,212,362	2	N_Shelf	1.16 × 10^−4^	−0.1932
cg09291474	*ANKDD1A*	65,223,141	15		1.21 × 10^−4^	0.1927
cg12682284	*C7orf31*	25,203,455	7		1.29 × 10^−4^	−0.1920
cg15791108	*intergenic*	2,653,571	17	S_Shore	1.46 × 10^−4^	0.1904
cg23707097	*PSD*	104,179,659	10	S_Shore	1.47 × 10^−4^	0.1903
cg04590841	*THBS2*	169,624,722	6	S_Shelf	1.48 × 10^−4^	−0.1903
cg19097359	*PPWD1*	64,859,901	5	S_Shore	1.49 × 10^−4^	−0.1902
cg17375585	*intergenic*	29,172,998	8		1.50 × 10^−4^	0.1901
cg13858602	*PUS1*	132,413,609	12	Island	1.57 × 10^−4^	0.1895
cg02139460	*COL28A1*	7,400,109	7		1.57 × 10^−4^	−0.1895

bp: base-pair (position is expressed in values from the Genome Reference Consortium Human Build 37 (GRCh37)-hg19). Chr: chromosome. *p*: *p*-value in the model adjusted for sex, age, diabetes, BMI, batch effect, smoking, and leukocyte cell-types. r: partial correlation coefficient for alcohol consumption. ^1^ Self-reported alcohol values (g/d) were transformed using the square root. ^2^ Relation to University of California-Santa Cruz genomic database (UCSC) according to its proximity to a CpG Island.

**Table 5:** Top 14 differentially methylated CpG sites filtered by their association with oxidative stress in the EWAS on self-reported alcohol ^1^ consumption ranked by smallest *p*-value after multivariable adjustment.

CpG	Gene Symbol	bp	Chr	*p*	r
cg05010179	*CYP1A1*	75,018,495	15	4.676 × 10^−3^	−0.1424
cg20983042	*IL1B*	113,594,963	2	1.026 × 10^−2^	−0.1294
cg19837601	*CYP2E1*	135,340,871	10	1.107 × 10^−2^	0.1280
cg27516584	*CYBA*	88,712,750	16	1.548 × 10^−2^	−0.1221
cg27008298	*ADH5*	100,010,193	4	1.562 × 10^−2^	0.1219
cg08845921	*ADH5*	100,011,210	4	1.820 × 10^−2^	−0.1191
cg10986462	*CYP2E1*	135,340,539	10	3.073 × 10^−2^	0.1090
cg10862468	*CYP2E1*	135,342,218	10	3.098 × 10^−2^	0.1088
cg25971823	*ACSS2*	33,515,143	20	3.405 × 10^−2^	−0.1069
cg08511598	*ALDH1B1*	38,392,771	9	3.629 × 10^−2^	−0.1057
cg20884605	*ALDH2*	112,205,368	12	3.955 × 10^−2^	0.1039
cg25163012	*SRXN1*	634,731	20	4.107 × 10^−2^	0.1031
cg16342538	*XDH*	31,607,367	2	4.134 × 10^−2^	0.1030
cg19571004	*CYP2E1*	135,340,850	10	4.461 × 10^−2^	0.1014

bp: base-pair (position is expressed in values from the GRCh37-hg19. Chr: chromosome. *p*: *p*-value in the model adjusted for sex, age, diabetes, BMI, batch effect, smoking, and leukocyte cell types. r: partial correlation coefficient for alcohol consumption. ^1^ Self-reported alcohol consumption (g/d) was transformed using the square root.

**Table 6:** Top 20 differentially methylated CpG sites identified in the EWAS on gamma-glutamyl transferase (GGT) levels ^1^ ranked by smallest *p*-value after multivariate adjustment.

CpG	Gene Symbol	bp	Chr	CpG Region ^2^	*p*	r
cg06690548	*SLC7A11*	139,162,808	4		3.03 × 10^−8^	−0.2756
cg05232694	*intergenic*	48,809,539	20	S_Shore	8.81 × 10^−8^	−0.2665
cg16033420	*GGT1*	25,015,744	22		3.47 × 10^−6^	−0.2322
cg09498080	*PKLR*	155,264,703	1	Island	4.55 × 10^−6^	−0.2295
cg19187075	*TRAF3IP2*	111,927,484	6		4.89 × 10^−6^	−0.2287
cg03973904	*intergenic*	3,409,242	18		5.03 × 10^−6^	−0.2285
cg13542857	*EP400NL*	132,600,670	12	N_Shore	8.78 × 10^−6^	−0.2227
cg13787154	*GGT1*	24,995,674	22		1.05 × 10^−5^	−0.2208
cg23280294	*RERE*	8,722,464	1		1.27 × 10^−5^	−0.2188
cg02499614	*PCDH1*	141,250,718	5		1.37 × 10^−5^	−0.2179
cg15275313	*C11orf36*	3,238,777	11	N_Shore	2.16 × 10^−5^	−0.2130
cg01251633	*intergenic*	122,914,607	10		2.28 × 10^−5^	0.2124
cg25416014	*MCF2L2*	183,146,809	3	S_Shore	3.14 × 10^−5^	0.2089
cg22099241	*MICA*	31,379,358	6		3.28 × 10^−5^	−0.2084
cg21830962	*UQCRC2*	21,964,544	16		3.77 × 10^−5^	0.2068
cg16399648	*COL27A1*	116,998,212	9		3.80 × 10^−5^	−0.2067
cg15769921	*TOP2A*	38,573,932	17	Island	3.89 × 10^−5^	0.2065
cg11005027	*GLB1L2*	134,237,890	11		4.23 × 10^−5^	−0.2055
cg26470501	*BCL3*	45,252,955	19	S_Shore	4.37 × 10^−5^	−0.2051
cg19693031	*TXNIP*	145,441,552	1		4.48 × 10^−5^	−0.2049

bp: base-pair (position is expressed in values from the GRCh37-hg19). Chr: chromosome. *p*: *p*-value in the model adjusted for sex, age, diabetes, BMI, batch effect, smoking, and leukocyte cell types. r: correlation coefficient for GGT. ^1^ GGT levels (U/L) were transformed using the natural logarithm. ^2^ Relation to UCSC CpG Island.

**Table 7:** Top 20 differentially methylated CpG sites identified in the EWAS on the epigenomic biomarker of alcohol ^1^ consumption ranked by smallest *p*-value after multivariate adjustment.

CpG	Gene Symbol	bp	Chr	CpG Region ^2^	*p*	r
cg06690548	*SLC7A11*	139,162,808	4		1.77 × 10^−23^	−0.4747
cg26457483	*PHGDH*	120,256,112	1	S_Shore	1.57 × 10^−12^	−0.3465
cg12825509	*TRA2B*	185,648,568	3		6.13 × 10^−12^	−0.3377
cg19731612	*NSD1*	176,559,334	5	Island	3.94 × 10^−9^	−0.2914
cg05856777	*SEC24B*	110,384,438	4		4.05 × 10^−9^	−0.2912
cg24911714	*MCRS1*	49,953,742	12		4.34 × 10^−9^	−0.2906
cg08021679	*NR5A2*	200,131,770	1		4.99 × 10^−9^	−0.2895
cg25027949	*intergenic*	22,897,581	14		6.49 × 10^−9^	−0.2875
cg21831333	*JPH4*	24,046,754	14	Island	8.60 × 10^−9^	0.2852
cg02661258	*FAM105B*	14,664,470	5	Island	8.76 × 10^−9^	0.2851
cg19782655	*SHANK2*	70,417,433	11		8.79 × 10^−9^	−0.2850
cg02921472	*C20orf30*	5,094,039	20	Island	9.84 × 10^−9^	0.2841
cg05681977	*SLC39A4*	145,638,934	8	Island	1.10 × 10^−8^	0.2833
cg04520581	*ALDH2*	112,204,510	12	Island	1.31 × 10^−8^	0.2818
cg00053261	*POM121L12*	53,103,214	7	N_Shore	1.32 × 10^−8^	−0.2817
cg19687075	*NOTCH1*	139,428,384	9	Island	1.40 × 10^−8^	0.2813
cg19681886	*PCMTD2*	62,902,916	20	S_Shore	1.50 × 10^−8^	−0.2807
cg18403220	*MRPS17*	56,019,978	7	Island	1.54 × 10^−8^	0.2805
cg09092356	*CISD1*	60,028,877	10	Island	1.58 × 10^−8^	0.2803
cg08000025	*MIR3134*	114,824,601	9		1.61 × 10^−8^	−0.2801

bp: base-pair (position is expressed in values from the GRCh37-hg19. Chr: chromosome). *p*: *p*-value in the model adjusted for sex, age, diabetes, BMI, batch effect, smoking, and leukocyte cell types. r: partial correlation coefficient for the epigenomic biomarker of alcohol consumption. ^1^ The epigenomic biomarker of alcohol consumption was computed using the 450-CpG based score. ^2^ Relation to UCSC CpG Island.

**Table 8:** Pathway enrichment of methylation of top-ranked sites, obtained in the epigenome-wide methylation analysis of the epigenomic score for alcohol intake in cohort 1, based on the Kyoto Encyclopedia of Genes and Genomes (KEGG). The pathway names are ranked according to the top 25 smallest *p*-values.

Pathway Name	Enrichment Score	Enrichment *p* ^1^	Bonferroni (Enrichment *p*) ^2^
Rap1 signaling pathway	19.3839	3.817 × 10^−9^	1.324 × 10^−6^
Pathways in cancer	18.495	9.284 × 10^−9^	3.221 × 10^−6^
Axon guidance	16.9057	4.549 × 10^−8^	1.579 × 10^−5^
Breast cancer	16.6497	5.877 × 10^−8^	2.039 × 10^−5^
MAPK signaling pathway	16.571	6.358 × 10^−8^	2.206 × 10^−5^
Regulation of actin cytoskeleton	16.5435	6.535 × 10^−8^	2.268 × 10^−5^
Pathways of neurodegeneration—mult. dis.	16.5329	6.604 × 10^−8^	2.292 × 10^−5^
Gastric cancer	15.0901	2.796 × 10^−7^	9.701 × 10^−5^
Phospholipase D signaling pathway	14.5526	4.785 × 10^−7^	1.660 × 10^−4^
Calcium signaling pathway	14.1153	7.410 × 10^−7^	2.571 × 10^−4^
Alzheimer disease	14.0186	8.162 × 10^−7^	2.832 × 10^−4^
Human papillomavirus infection	13.8919	9.264 × 10^−7^	3.215 × 10^−4^
mTOR signaling pathway	13.7378	1.081 × 10^−6^	3.751 × 10^−4^
Platelet activation	13.4365	1.461 × 10^−6^	5.069 × 10^−4^
Cushing syndrome	12.8227	2.699 × 10^−6^	9.364 × 10^−4^
Spinocerebellar ataxia	12.296	4.570 × 10^−6^	1.586 × 10^−3^
Hepatocellular carcinoma	12.2638	4.720 × 10^−6^	1.638 × 10^−3^
Basal cell carcinoma	12.0647	5.759 × 10^−6^	1.998 × 10^−3^
Focal adhesion	11.8482	7.152 × 10^−6^	2.482 × 10^−3^
Sphingolipid signaling pathway	11.8093	7.435 × 10^−6^	2.580 × 10^−3^
Hippo signaling pathway	11.5789	9.361 × 10^−6^	3.248 × 10^−3^
p53 signaling pathway	11.1705	1.408 × 10^−5^	4.887 × 10^−3^
Metabolic pathways	11.0965	1.517 × 10^−5^	5.263 × 10^−3^
Endocytosis	11.0703	1.557 × 10^−5^	5.402 × 10^−3^
Endocrine resistance	10.9624	1.734 × 10^−5^	6.018 × 10^−3^

^1^ *p*-value for the pathway enrichment based on KEGG. ^2^ *p*-value for the Bonferroni correction.

**Table 9:** Association between the epigenomic biomarker of alcohol consumption and selected biomarkers of aging.

Biomarker	Beta ^1^ (SE ^1^)	*p* ^1^	*p* ^2^	*p* ^3^
DNAm telomere length	−0.259 (0.045)	<0.001	<0.001	<0.001
GrimAgeAccel	0.243 (0.039)	<0.001	<0.001	<0.001
PhenoAgeAccel	0.250 (0.048)	<0.001	<0.001	<0.001
CausAgeYing	0.286 (0.042)	<0.001	<0.001	<0.001

^1^ Regression model adjusted for sex, age, diabetes, BMI, and smoking; ^2^ model 1 additionally adjusted for physical activity, medications, and educational level; ^3^ model 2 additionally adjusted for adherence to Mediterranean diet; biomarkers were scaled using Z-transformation.

## Data Availability

Neither the participants’ consent forms nor ethics approval included permission for open access. However, we follow a controlled data-sharing collaboration model, and data for collaborations will be available upon request pending application and approval. Investigators who are interested in this study can contact the corresponding author.

## Supplementary Materials

The following supporting information can be downloaded at: https://www.mdpi.com/article/10.3390/antiox15020197/s1, Table S1: Annotated CpG sites for the computation of the 450 CpGs-epigenomic score for alcohol consumption. Top 15 CpG sites (positive weights) and bottom 15 CpG sites (negative weights) were listed; Figure S1: Overview of the main characteristics, data obtained and analyses conducted in the main cohort (1) and in the replication cohort (2); Figure S2: Box plot of the levels of alcohol consumption according to the international cut-off in men and women; Figure S3: Descriptives for the epigenomic biomarker of alcohol consumption in cohort 1: (a) frequency distribution of the epigenomic score for alcohol consumption; (b) correlation between platforms; Figure S4: Box plots for the association between the biomarkers of alcohol consumption and the self-reported alcohol intake in cohort 1, grouped in 4 categories, using the standard drinks cut-off levels for the Spanish; Figure S5: Scatter plots and regression lines for the relationship between: (a) the plasma GGT levels and the self-reported alcohol intake; (b) the epigenomic biomarker of alcohol intake and the self-reported alcohol consumption in drinkers (cohort 1); Figure S6: Scatter plot and regression line for the relationship between the epigenomic biomarker of alcohol intake and the self-reported alcohol consumption in drinkers (cohort 2); Table S2: Gene enrichment results for the most significant methylation sites (FDR < 0.05) in the epigenome-wide methylation analysis for epigenomic alcohol carried out using the gene ontology (GO) platform; Figure S7: Associations between self-reported alcohol consumption (4 categories) and biomarkers of aging in cohort 1; Figure S8: Scatter plots and regression lines for the association between the epigenomic biomarker of alcohol intake and the selected clocks in cohort 1: (a) DNAm telomere length adjusted age; (b) GrimAgeAcceleration; Figure S9: Scatter plots and regression lines for the association between the epigenomic biomarker of alcohol intake and the selected clocks in cohort 2: (a) DNAm telomere length adjusted age; (b) GrimAgeAcceleration; Figure S10: DNAm telomere length depending on the self-reported alcohol intake (4 categories) and adherence to the Mediterranean diet (MedDiet); Figure S11: DNAm telomere length in cohort 1 depending on the self-reported alcohol intake (2 categories: non-drinkers + light drinkers; moderate + heavy drinkers) and levels of adherence to the Mediterranean diet; Table S3: Analysis of the potential overlap among CpGs included in the epigenomic score for alcohol consumption, and the CpGs included in the selected biomarkers of aging. References [63,77] are cited in the Supplementary Materials.
