# Supplementary material for: Alcohol Consumption and DNA Methylation in a Mediterranean Cohort: A Focus on Oxidative Stress and Aging Biomarkers
Source: Antioxidants (Basel). 2026 Feb 2;15(2):197. doi: 10.3390/antiox15020197 (PMC12938750; doi:10.3390/antiox15020197)
Supplement: Supplementary file 1 [file antioxidants-15-00197-s001.zip › antioxidants-4070372-supplementary.pdf]

Article

Alcohol consumption and DNA-methylation in a Mediterranean cohort: focus on oxidative stress and aging biomarkers

Oscar Coltell, Eva. M. Asensio, José V. Sorlí, Rebeca Fernández-Carrión, Carolina Ortega-Azorín, Rocío Barragán, Alejandro Perez-Fidalgo, Olga Portolés, José M. Ordovás, and Dolores Corella

SUPPLEMENTARY MATERIALS

Index

Table S1. Annotated CpG sites for the computation of the 450 CpGs-epigenomic score for alcohol consumption. Top 15 CpG sites (positive weights) and bottom 15 CpG sites (negative weights) were listed.

Figure S1. Overview of the main characteristics, data obtained and analyses conducted in the main cohort (1) and in the replication cohort (2).

Figure S2. Box plot of the levels of alcohol consumption according to the international cut-off previously reported by Liu et al. [1] in men and women.

Figure S3. Descriptives for the epigenomic biomarker of alcohol consumption in cohort 1: (a) Frequency distribution of the epigenomic score for alcohol consumption; (b) Correlation between platforms.

Figure S4. Box plots for the association between the biomarkers of alcohol consumption and the self-reported alcohol intake in cohort 1, grouped in 4 categories, using the standard drinks cut-off levels for the Spanish.

Figure S5. Scatter plots and regression lines for the relationship between: (a) The plasma GGT levels and the self-reported alcohol intake; (b) The epigenomic biomarker of alcohol intake and the self-reported alcohol consumption in drinkers (cohort 1).

Figure S6. Scatter plot and regression line for the relationship between the epigenomic biomarker of alcohol intake and the self-reported alcohol consumption in drinkers (cohort 2).

Table S2. Gene enrichment results for the most significant methylation sites (FDR < 0.05) in the epigenome-wide methylation analysis for epigenomic alcohol carried out using the gene ontology (GO) platform.

Figure S7. Associations between self-reported alcohol consumption (4 categories) and biomarkers of aging in cohort 1.

Figure S8. Scatter plots and regression lines for the association between the epigenomic biomarker of alcohol intake and the selected clocks in cohort 1: (a) DNAm Telomere length adjusted age; (b) GrimAgeAcceleration.

Figure S9. Scatter plots and regression lines for the association between the epigenomic biomarker of alcohol intake and the selected clocks in cohort 2: (a) DNAm Telomere length adjusted age; (b) GrimAgeAcceleration.

Figure S10. DNAm telomere length depending on the self-reported alcohol intake (4 categories) and adherence to the Mediterranean diet (MedDiet).

Figure S11. DNAm telomere length in cohort 1depending on the self-reported alcohol intake (2 categories: non-drinkers + light drinkers; moderate + heavy drinkers) and levels of adherence to the Mediterranean diet.

Table S3. Analysis of the potential overlap among CpGs included in the epigenomic score for alcohol consumption, and the CpGs included in the selected biomarkers of aging.

Working Index

Table S1. Annotated CpG sites for the computation of the 450 CpGs-epigenomic score for alcohol consumption. Top 15 CpG sites (positive weights) and bottom 15 CpG sites (negative weights) were listed. The full list (only indicating CpG names and Beta values) was published by McCartney et al. [2].

Figure S1. Overview of the main characteristics, data obtained and analyses conducted in the main cohort (1) and in the replication cohort (2).

Figure S2. Box plot of the levels of alcohol consumption according to the international cut-off previously reported by Liu et al. [1] in men and women: Non-drinkers (alcohol = 0); light drinkers (≤ 28 g/d in men and ≤ 14 g/d in women); "at risk drinkers" (28-42 g/d in men and 14-28 g/d in women); and heavy drinkers (≥ 42 g/d in men and ≥ 28 g/d in women). In this population, these figures were: 100, 271, 28 and 15 respectively, in cohort 1.

Figure S3. Descriptives for the epigenomic biomarker of alcohol consumption in cohort 1: (**a**) Frequency distribution of the epigenomic score for alcohol consumption in this sample (n=414); (**b**) Perfect correlation (r^2=1) between the epigenomic score computed in platform 1 (UCLA) and platform 2 (Biolearn).

Figure S4. Box plots for the association between the biomarkers of alcohol consumption and the self-reported alcohol intake in cohort 1, grouped in 4 categories, using the standard drinks cut-off levels for the Spanish population as indicated in methods: (**a**) Association with the GGT plasma levels and the corresponding p-value; (**b**) Association with the epigenomic biomarker and the corresponding p-value.

Figure S5. Scatter plots and regression lines for the relationship between: (**a**) The plasma GGT levels (ln) and the self-reported alcohol intake in drinkers (in g/d, square-root-transformed) (cohort 1); (**b**) The epigenomic biomarker of alcohol intake (in g/d, z-transformed) and the self-reported alcohol consumption in drinkers (cohort 1). r correlation coefficient and the corresponding p-values are presented.

Figure S6. Scatter plot and regression line for the relationship between the epigenomic biomarker of alcohol intake (in g/d, z-transformed) (cohort 2) and the self-reported alcohol intake in drinkers (in g/d, square-root-transformed). r correlation coefficient and the corresponding p-value are presented.

Table S2. Gene enrichment results for the most significant methylation sites (FDR < 0.05) in the epigenome-wide methylation analysis for epigenomic alcohol[1] carried out using the gene ontology (GO) platform. The GO function, type, enrichment score, enrichment p-value and the Bonferroni corrected p-value are presented for the top 30 functions ranked by the smallest p-values.

Figure S7. Associations between self-reported alcohol consumption (4 categories) and biomarkers of aging in cohort 1: (**a**) With GrimAgeAcceleration; (**b**) With PhenoAgeAcceleration. Biomarkers were z-transformed, and models were adjusted for: (1) sex., age, diabetes, BMI and smoking; (2) model 1 additionally adjusted for physical activity, medications and education. Error bars: SE of means.

Figure S8. Scatter plots and regression lines for the association between the epigenomic biomarker of alcohol intake and the selected clocks in cohort 1: (**a**) DNAm Telomere length adjusted age (z-transformed); (**b**) GrimAgeAcceleration (z-transformed). r correlation coefficient and the corresponding p-values are presented.

Figure S9. Scatter plots and regression lines for the association between the epigenomic biomarker of alcohol intake and the selected clocks in cohort 2: (**a**) DNAm Telomere length adjusted age (z-transformed); (**b**) GrimAgeAcceleration (z-transformed). r correlation coefficient and the corresponding p-values are presented.

Figure S10. DNAm telomere length (adjusted for age and z-score transformed) depending on the self-reported alcohol intake (4 categories) and adherence to the Mediterranean diet (MedDiet), based on the MEDAS-17 in cohort 1after

excluding wine intake (maximum 16 points). Low (0-8) and High (9-16) levels were considered. Regression model was adjusted for sex, age, diabetes, BMI and smoking. P-value for the interaction term is indicated in this model. Error bars: SE of means.

Figure S11. DNAm telomere length (adjusted for age and z-score transformed) in cohort 1depending on the self-reported alcohol intake (2 categories: non-drinkers + light drinkers; moderate + heavy drinkers) and levels of adherence to the Mediterranean diet (MedDiet) after excluding wine intake in MEDAS-17: Low (0-8) and High (9-16). Regression models were adjusted for: (1) sex, age, diabetes, BMI and smoking; (2) additionally adjusted for physical activity, medications and education. P-values for the interaction terms are indicated for each model. Error bars: SE of means.

Table S3. Analysis of the potential overlap among CpGs included in the epigenomic score for alcohol consumption (the full list of 450 CpGs was published by McCartney et al. [2]), and the CpGs included in the selected biomarkers of aging: (a) List of overlapping CpGs; (b) Gene symbol and chromosome of the overlapped CpGs.

Table S1. Annotated CpG sites for the computation of the 450 CpGs-epigenomic score for alcohol consumption. Top 15 CpG sites (positive weights) and bottom 15 CpG sites (negative weights) were listed. The full list (only indicating CpG names and Beta values) was published by McCartney et al. [2].

CpG	Beta [1]	Chr	Gene symbol	bp	CpG group [2]
cg16113793	3.8626	18	LAMA3	21451607	TSS1500;TSS1500;Body;Body
cg15970375	2.5283	11	ZBTB44	130184946	TSS1500
cg16057915	2.1661	1	PEX14	10690564	3'UTR
cg01503881	1.7681	20	ATP5E	57607569	TSS200;TSS200
cg02650017	1.6552	17	PHOSPHO1	47301614	Body;Body
cg08174504	1.3851	4	ANTXR2	80993819	5'UTR;1stExon;1stExon;5'UTR
cg19613400	1.1948	1	BMP8B	40254712	TSS200
cg15537269	1.0830	8		24507197	
cg08033031	0.9931	19	EPS15L1	16528764	Body
cg27078522	0.9852	7	KIAA0415	4815167	TSS200
cg15804767	0.9295	6	LTB	31548596	Body;3'UTR
cg27118035	0.9087	16	ZNF267	31891978	Body
cg03440556	0.7988	10	SCD	102107757	Body
cg05926784	0.7959	19	RFX1	14073277	3'UTR
cg24011637	0.7666	2	FOSL2	28614567	TSS1500
cg26008007	-0.9173	2		232545866	
cg19692318	-0.9414	6	EPB41L2	131384600	TSS200;TSS200;TSS200
cg12477533	-0.9610	2	VWA3B	98703450	TSS200
cg24537348	-1.0144	17	FOXK2	80545705	Body
cg07814318	-1.0309	15	KLF13	31624584	Body
cg19706803	-1.0633	1	PRDM16	3126033	Body;Body
cg06516445	-1.1488	11	DCUN1D5	102963032	TSS200
cg18917643	-1.1516	5		39476124	
cg06523224	-1.3606	15	BNC1	83953883	TSS1500
cg12825509	-1.4181	3	TRA2B	185648568	Body
cg05962079	-1.5133	1	PLCH2	2428333	Body
cg24429337	-1.7024	2	IL1F7	113676413	3'UTR;3'UTR;3'UTR;3'UTR;3'UTR
cg20624830	-1.8003	10		22725885	
cg06690548	-2.5993	4	SLC7A11	139162808	Body
cg01305596	-5.0428	16	ABCC1	16228299	Body;Body;Body;Body;Body

bp: Base-pair. Chr: Chromosome. [1] Beta value reported by McCartney et al. [2] in the published score; [2] Univer-sity of California-Santa Cruz genomic database (UCSC) CpG group that classifies the location of a CpG site in relation to the structure of regions in the corresponding gene.

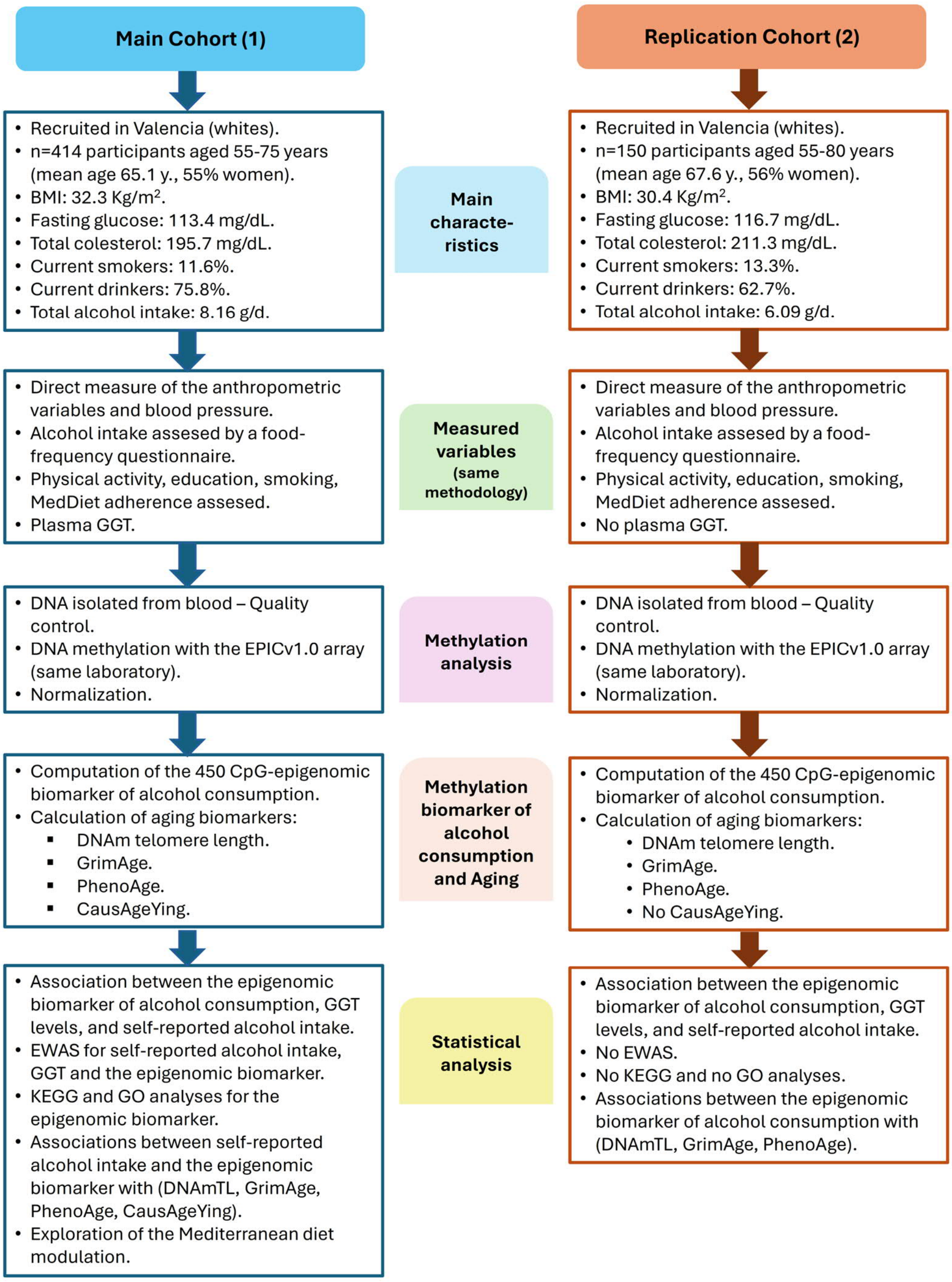

Figure S1. Overview of the main characteristics, data obtained and analyses conducted in the main cohort (1) and in the replication cohort (2).

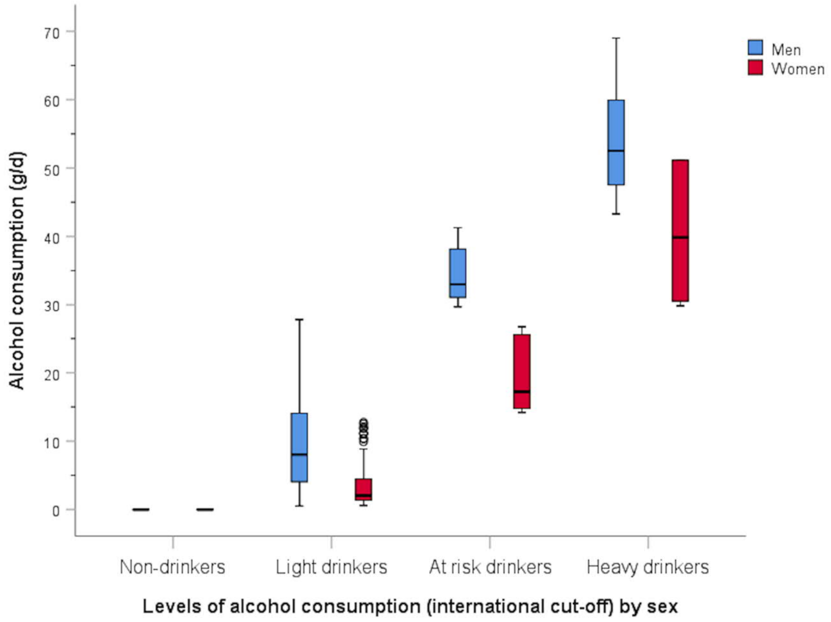

Figure S2. Box plot of the levels of alcohol consumption according to the international cut-off previously reported by Liu et al. [1] n men and women: Non-drinkers (alcohol = 0); light drinkers (≤ 28 g/d in men and ≤ 14 g/d in women); “at risk drinkers” (28-42 g/d in men and 14-28 g/d in women); and heavy drinkers (≥ 42 g/d in men and ≥ 28 g/d in women). In this popula-tion, these figures were: 100, 271, 28 and 15 respectively, in cohort 1.

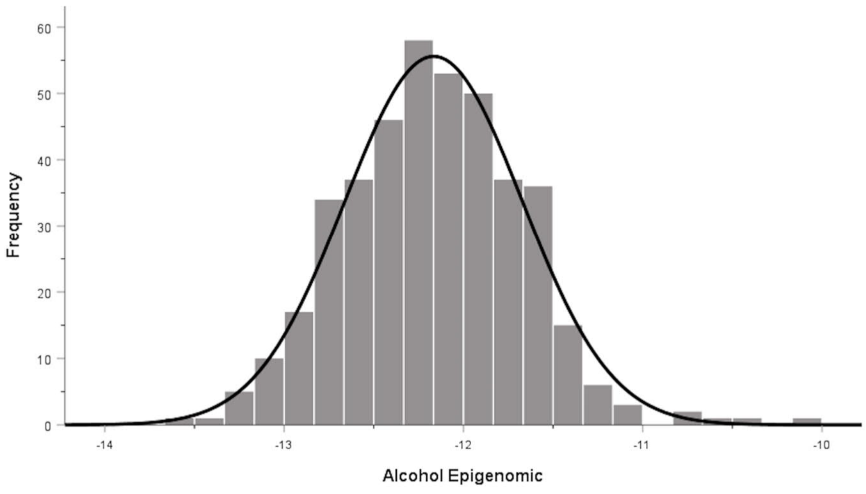

(**a**)

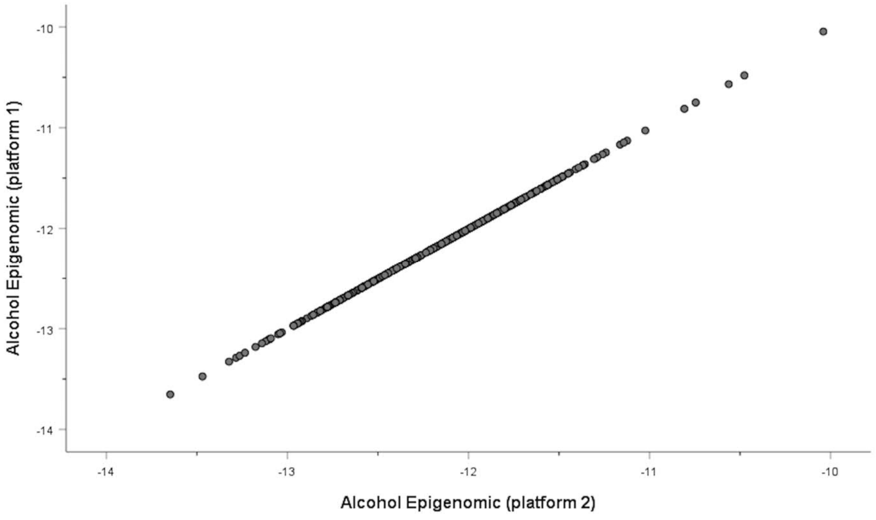

(**b**)

Figure S3. Descriptives for the epigenomic biomarker of alcohol consumption in cohort 1: (**a**) Frequency distribution of the epigenomic score for alcohol consumption in this sample (n = 414); (**b**) Perfect correlation ($r^2 = 1$) between the epigenomic score computed in platform 1 (UCLA) and platform 2 (Biolearn).

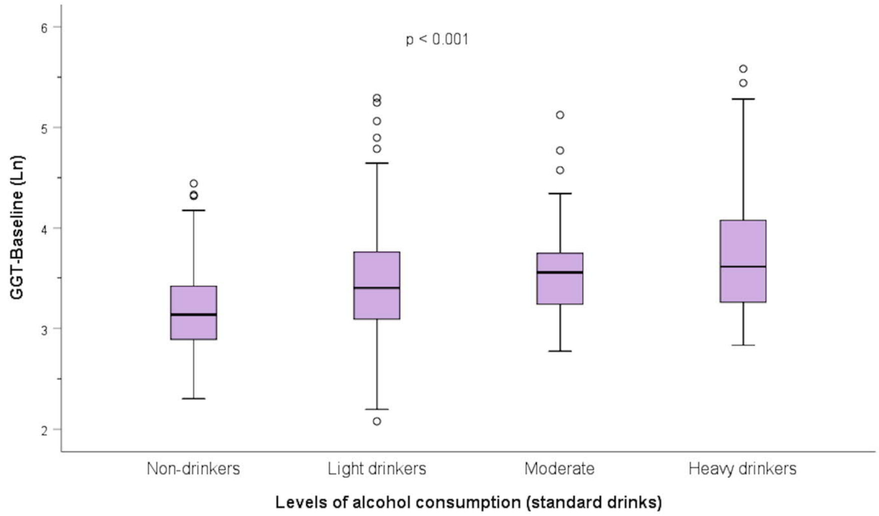

(**a**)

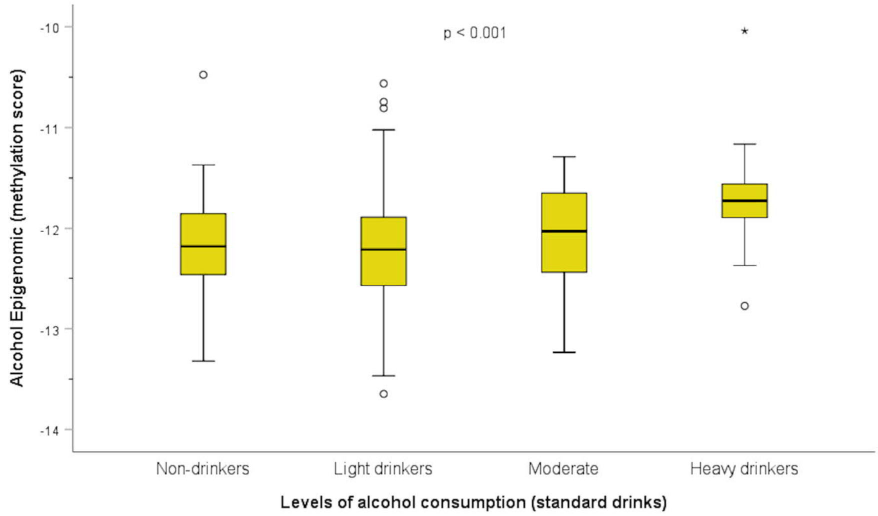

(**b**)

Figure S4. Box plots for the association between the biomarkers of alcohol consumption and the self-reported alcohol intake in cohort 1, grouped in 4 categories, using the standard drinks cut-off levels for the Spanish population as indicated in methods: (**a**) Association with the GGT plasma levels and the corresponding p-value; (**b**) Association with the epigenomic biomarker and the corresponding p-value.

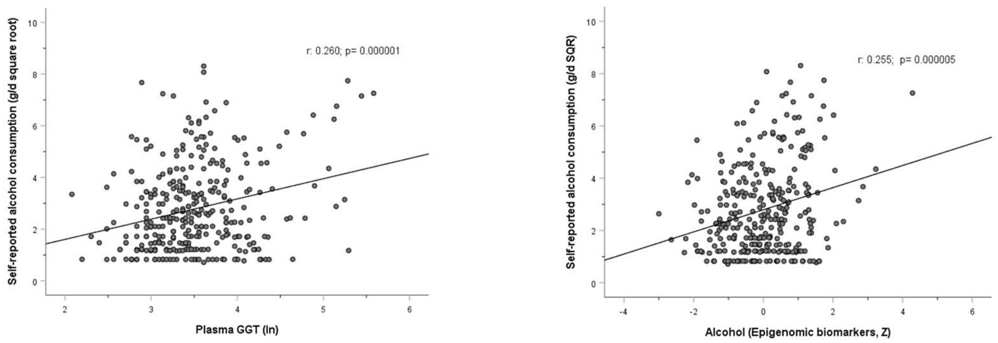

(**a**) (**b**)

Figure S5. Scatter plots and regression lines for the relationship between: (**a**) The plasma GGT levels (ln) and the self-reported alcohol intake in drinkers (in g/d, square-root-transformed) (cohort 1); (**b**) The epigenomic biomarker of alcohol intake (in g/d, z-transformed) and the self-reported alcohol consumption in drinkers (cohort 1). r correlation coefficient and the corresponding p-values are presented.

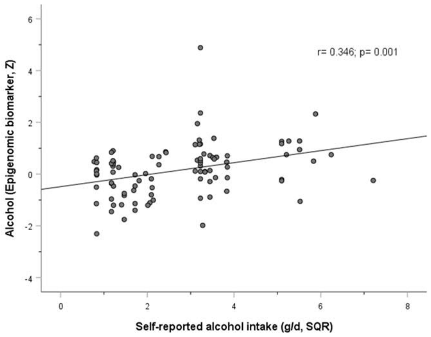

Figure S6. Scatter plot and regression line for the relationship between the epigenomic biomarker of alcohol intake (in g/d, z-transformed) (cohort 2) and the self-reported alcohol intake in drinkers (in g/d, square-root-transformed). r correlation coefficient and the corresponding p-value are presented.

Table S2. Gene enrichment results for the most significant methylation sites (FDR < 0.05) in the epigenome-wide methylation analysis for epigenomic alcohol[1] carried out using the gene ontology (GO) platform. The GO function, type, enrichment score, enrichment p-value and the Bonferroni corrected p-value are presented for the top 30 functions ranked by the smallest p-values.

GO Function	type	Enrichment Score	Enrichment p	Bonferroni (Enrichment p)
organelle	cellular component	183.747	1.584×10^{-80}	2.290×10^{-76}
protein binding	molecular function	171.004	5.418×10^{-75}	7.832×10^{-71}
intracellular organelle	cellular component	150.23	5.700×10^{-66}	8.240×10^{-62}
binding	molecular function	139.323	3.111×10^{-61}	4.497×10^{-57}
membrane-bounded organelle	cellular component	127.174	5.875×10^{-56}	8.493×10^{-52}
regulation of signaling	biological process	119.845	8.953×10^{-53}	1.294×10^{-48}
regulation of cell communication	biological process	118.804	2.536×10^{-52}	3.667×10^{-48}
cytosol	cellular component	112.147	1.973×10^{-49}	2.852×10^{-45}
positive regulation of cellular process	biological process	109.622	2.465×10^{-48}	3.563×10^{-44}
positive regulation of biological process	biological process	108.669	6.390×10^{-48}	9.238×10^{-44}
intracellular membrane-bounded organelle	cellular component	105.93	9.890×10^{-47}	1.430×10^{-42}
protein-containing complex	cellular component	104.725	3.299×10^{-46}	4.769×10^{-42}
negative regulation of cellular process	biological process	104.391	4.608×10^{-46}	6.661×10^{-42}
organic cyclic compound binding	molecular function	103.148	1.597×10^{-45}	2.308×10^{-41}
regulation of signal transduction	biological process	98.1575	2.348×10^{-43}	3.395×10^{-39}
negative regulation of biological process	biological process	97.3352	5.344×10^{-43}	7.725×10^{-39}
cellular component organization or biogenesis	biological process	94.643	7.890×10^{-42}	1.141×10^{-37}
cellular component organization	biological process	93.4574	2.582×10^{-41}	3.733×10^{-37}
cellular process	biological process	87.2848	1.238×10^{-38}	1.790×10^{-34}
regulation of metabolic process	biological process	81.1655	5.627×10^{-36}	8.134×10^{-32}
regulation of primary metabolic process	biological process	79.1977	4.026×10^{-35}	5.820×10^{-31}
nucleoplasm	cellular component	76.4431	6.327×10^{-34}	9.146×10^{-30}
regulation of cellular component organization	biological process	75.7562	1.257×10^{-33}	1.817×10^{-29}
positive regulation of macromolecule metabolic process	biological process	74.4637	4.579×10^{-33}	6.620×10^{-29}
regulation of cellular metabolic process	biological process	73.7725	9.141×10^{-33}	1.321×10^{-28}
regulation of macromolecule metabolic process	biological process	73.387	1.344×10^{-32}	1.942×10^{-28}
positive regulation of metabolic process	biological process	72.4559	3.410×10^{-32}	4.929×10^{-28}
regulation of response to stimulus	biological process	71.9515	5.647×10^{-32}	8.164×10^{-28}
positive regulation of nucleobase-containing compound metabolic process	biological process	67.3474	5.641×10^{-30}	8.155×10^{-26}
adenyl nucleotide binding	molecular function	64.8358	6.953×10^{-29}	1.005×10^{-24}

[1]: The epigenomic score for alcohol consumption was used as a continuous variable and regression models were adjusted for sex, age, diabetes, BMI, batch effect, smoking and leukocyte cell levels in the corresponding EWAS. FDR: False Discovery Rate.

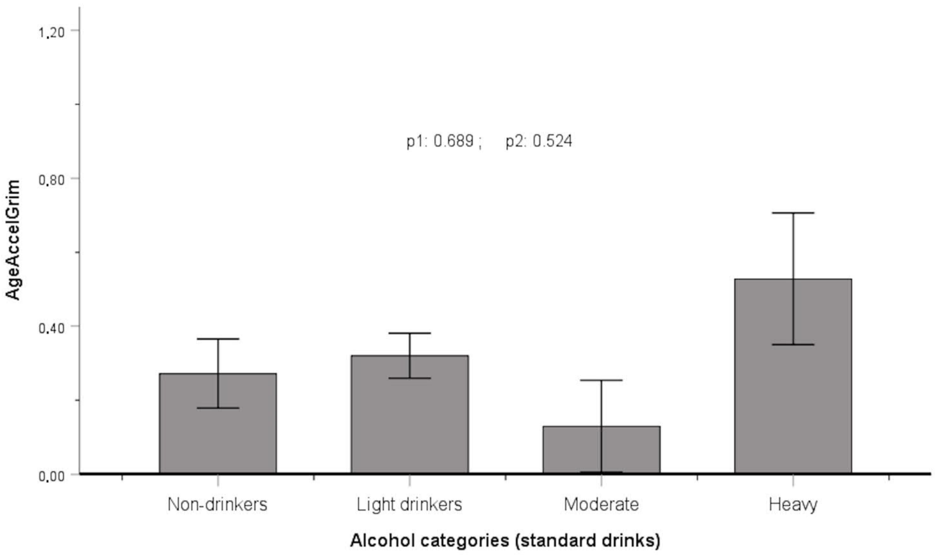

(**a**)

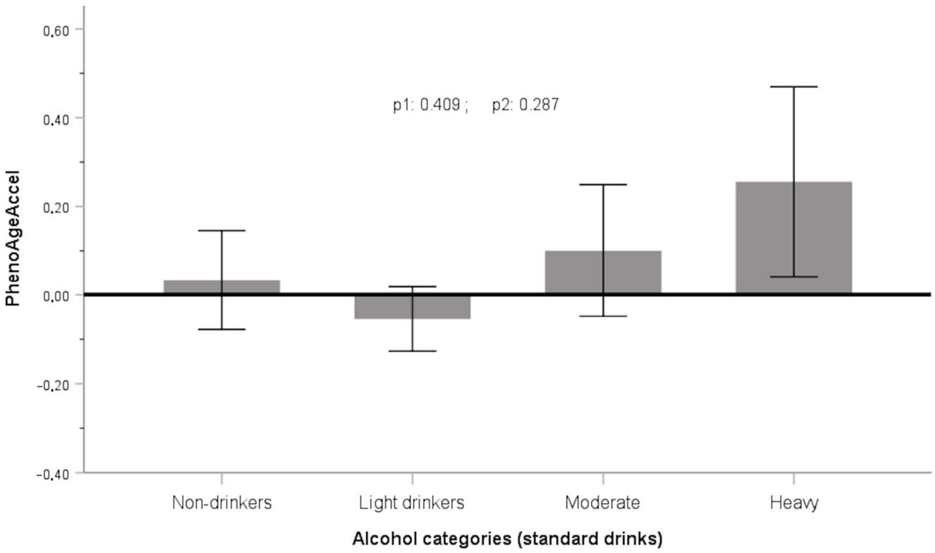

(**b**)

Figure S7. Associations between self-reported alcohol consumption (4 categories) and biomarkers of aging in cohort 1: (**a**) With GrimAgeAcceleration; (**b**) With PhenoAgeAcceleration. Biomarkers were z-transformed, and models were adjusted for: (1) sex., age, diabetes, BMI and smoking; (2) model 1 additionally adjusted for physical activity, medications and education. Error bars: SE of means.

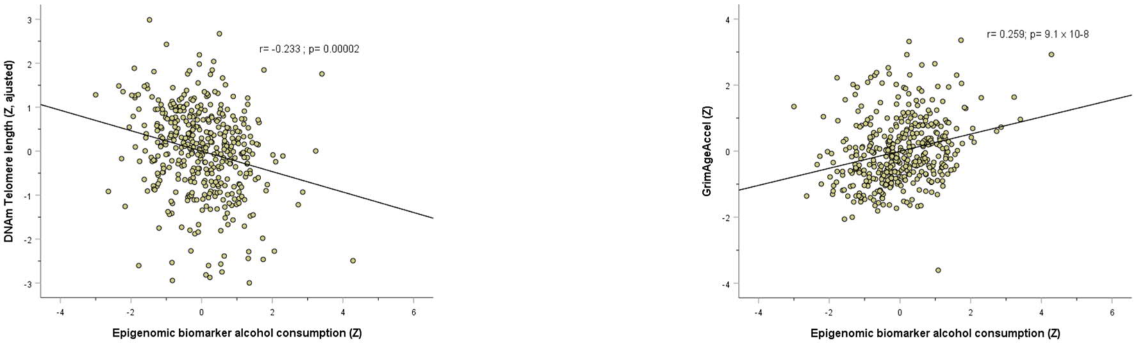

(**a**) (**b**)

Figure S8. Scatter plots and regression lines for the association between the epigenomic biomarker of alcohol intake and the selected clocks in cohort 1: (**a**) DNAm Telomere length adjusted age (z-transformed); (**b**) GrimAgeAcceleration (z-transformed). r correlation coefficient and the corresponding p-values are presented.

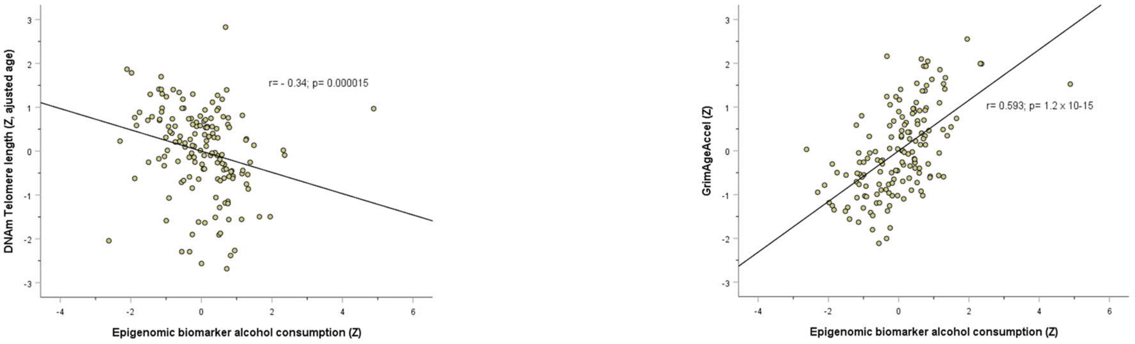

(**a**) (**b**)

Figure S9. Scatter plots and regression lines for the association between the epigenomic biomarker of alcohol intake and the selected clocks in cohort 2: (**a**) DNAm Telomere length adjusted age (z-transformed); (**b**) GrimAgeAcceleration (z-transformed). r correlation coefficient and the corresponding p-values are presented.

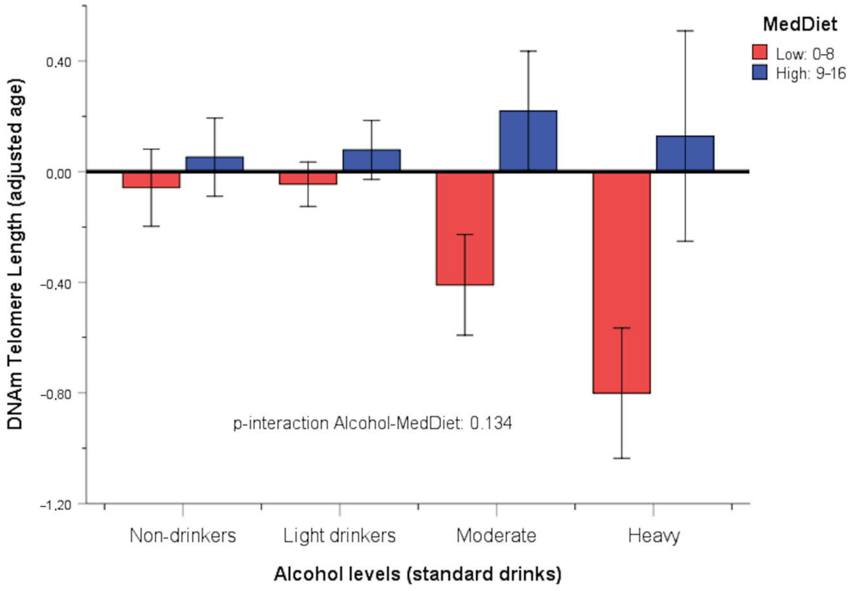

Figure S10. DNAm telomere length (adjusted for age and z-score transformed) depending on the self-reported alcohol intake (4 categories) and adherence to the Mediterranean diet (MedDiet), based on the MEDAS-17 in cohort 1after excluding wine intake (maximum 16 points). Low (0-8) and High (9-16) levels were considered. Regression model was adjusted for sex, age, diabetes, BMI and smoking. P-value for the interaction term is indicated in this model. Error bars: SE of means.

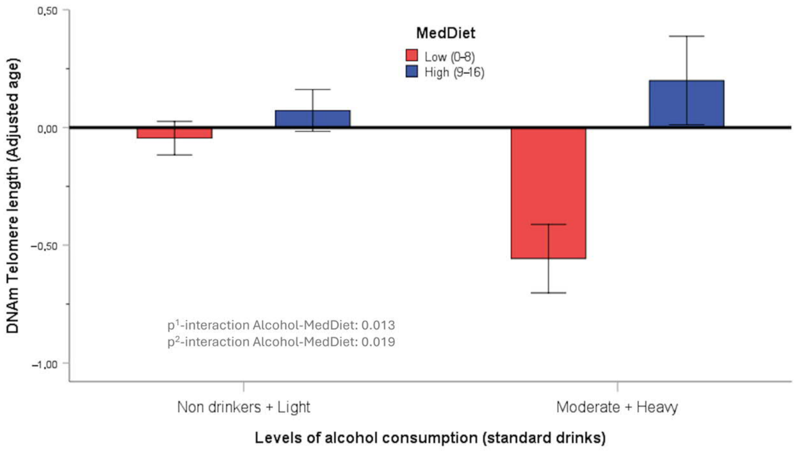

Figure S11. DNAm telomere length (adjusted for age and z-score transformed) in cohort 1depending on the self-reported alcohol intake (2 categories: non-drinkers + light drinkers; moderate + heavy drinkers) and levels of adherence to the Mediterranean diet (MedDiet) after excluding wine intake in MEDAS-17: Low (0-8) and High (9-16). Regression models were adjusted for: (1) sex, age, diabetes, BMI and smoking; (2) additionally adjusted for physical activity, medications and education. P-values for the interaction terms are indicated for each model. Error bars: SE of means.

Table S3. Analysis of the potential overlap among CpGs included in the epigenomic score for alcohol consumption (the full list of 450 CpGs was published by McCartney et al. [2]), and the CpGs included in the selected biomarkers of aging: (a) List of overlapping CpGs; (b) Gene symbol and chromosome of the overlapped CpGs.

(a) Biomarker of aging	CpGs in biomarker of aging	Total	Over-lapped CpGs	Diffe rent CpGs	IDs for overlapped CpGs
DNAmTL	141	450	0	450	none
GrimAgeV1	1141	450	5	445	cg01412762, cg17886420, cg04832325, cg14476101, cg06690548
PhenoAge	514	450	1	449	cg06690548
YingCausAge	586	450	1	449	cg21154793

(b) CpG-Biomarker	Overlapped CpG	Gene Symbol	Chr
cg01412762_GrimAgeV1	cg01412762	ALK	2
cg04832325_GrimAgeV1	cg04832325	KIRREL3	11
cg06690548_GrimAgeV1	cg06690548	SLC7A11	4
cg14476101_GrimAgeV1	cg14476101	PHGDH	1
cg17886420_GrimAgeV1	cg17886420	intergenic	10
cg06690548_PhenoAge	cg06690548	SLC7A11	4
cg21154793_YingCausAge	cg21154793	CACNG6	19

Chr: Chromosome.

References

1. Liu, C.; Marioni, R.E.; Hedman, Å.K.; Pfeiffer, L.; Tsai, P.-C.; Reynolds, L.M.; Just, A.C.; Duan, Q.; Boer, C.G.; Tanaka, T.; et al. A DNA Methylation Biomarker of Alcohol Consumption. *Mol. Psychiatry* **2018**, *23*, 422–433. https://doi.org/10.1038/mp.2016.192.
2. McCartney, D.L.; Hillary, R.F.; Stevenson, A.J.; Ritchie, S.J.; Walker, R.M.; Zhang, Q.; Morris, S.W.; Bermingham, M.L.; Campbell, A.; Murray, A.D.; et al. Epigenetic Prediction of Complex Traits and Death. *Genome Biol.* **2018**, *19*, 136. https://doi.org/10.1186/s13059-018-1514-1.
